# What Other Than Acridinium Esters? Computational Search for New Acridinium-Based Chemiluminogens

**DOI:** 10.3390/ijms24119468

**Published:** 2023-05-30

**Authors:** Milena Pieńkos, Beata Zadykowicz

**Affiliations:** Faculty of Chemistry, University of Gdańsk, Wita Stwosza 63, 80-308 Gdańsk, Poland; milena.pienkos@phdstud.ug.edu.pl

**Keywords:** DFT and TD DFT calculations, chemiluminescence reaction, acridinium derivatives, design of chemiluminogen

## Abstract

The rapid increase in disease prevalence in the world makes it extremely important to search for new or develop existing diagnostic methods, for example, chemiluminescent labeling used in immunodiagnostics. At present, acridinium esters are willingly used as chemiluminogenic fragments of labels. However, the search for new chemiluminogens that are particularly efficient is the main task of our studies. The density functional theory (DFT) and time-dependent (TD) DFT methods were used to obtain thermodynamic and kinetic results concerning the chemiluminescence and competitive dark reactions, which indicated whether some of the scrutinized derivatives have better characteristics than the chemiluminogens used so far. Synthesis of these candidates for efficient chemiluminogens, followed by studies of their chemiluminescent properties, and ultimately in chemiluminescent labeling, are further steps to confirm their potential applicability in immunodiagnostics.

## 1. Introduction

Chemiluminogens are chemical compounds with surprising properties. These compounds can react with certain substances to form products or intermediate products in an electronically excited state [1,2]. These electronically excited products returning to the ground state can transfer excess energy in the form of light emission [2]. Therefore, it can be said that chemiluminogens are substances that undergo chemical reactions to create light. Many chemical compounds have chemiluminogenic properties, including organic (e.g., luminol [1,3] acridinium derivatives [4,5,6,7,8,9,10,11], lucigenin [12,13], oxalic acid [14,15] derivatives), and inorganic compounds (e.g., white phosphorus [16,17], chloric acid salts [18]). However, mainly organic chemiluminogens have several applications, especially as chemiluminescent indicators [19] and chemiluminogenic fragments of chemiluminescent labels [20]. One of the groups of compounds that has found numerous applications in medical [21,22], chemical [2,23], biochemical [24], and environmental [25,26] analysis are acridine derivatives, especially acridinium esters [2,4,5,26,27]. This group of chemiluminogens is characterized by high chemiluminescence efficiency (reaching several percent vs. ~1.3% obtained with luminol) [4,27,28] without the necessity of using a catalyst to initiate chemiluminescence and a low level of background [29]. Additionally, these compounds are easily synthesized [4,5,8,30]. Simultaneously, using acridinium esters enable a determination of the concentrations of biological entities such as antigens [31], antibodies [32,33], hormones [21,22], fragments of nucleic acids [34], and others at the ultra-sensitive level [35] (limits of detection at the level of 10^−19^ mole of analyte or below).

**Scheme 1:**
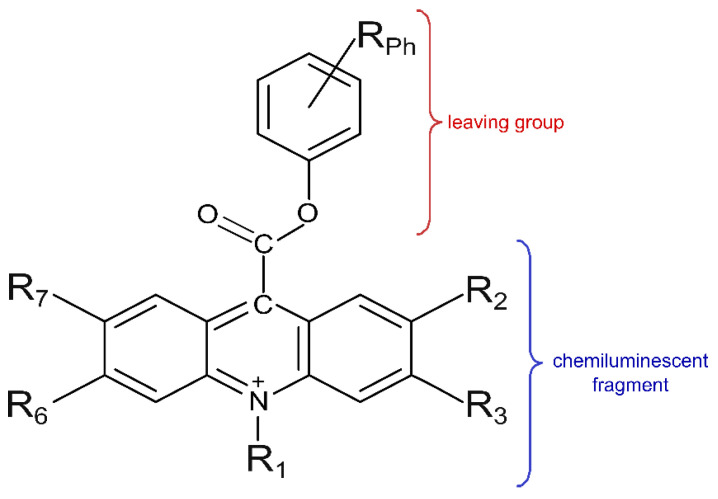
The chemical structure of 10-methyl-9-(phenoxycarbonyl)acridinium (acridinium ester) cations.

As previous studies have shown [4,5,6,7,36], acridinium derivatives are easily oxidized with hydrogen peroxide in an alkaline environment. These compounds are susceptible to nucleophilic attack on the carbon atom at the 9 position of acridinium moiety. In the next step, the reaction with hydroxide anion follows, and as a result, an energy-rich cyclic intermediate arises. In the end, the cyclic intermediate decomposes, by which an electronically excited product—the molecule of 10-methyl-9-acridinone [6,7,36,37]—is formed. From the point of view of the mechanism of chemiluminescence reaction and easier formation of electronically excited products, acridinium derivatives may be accordingly modified. The substitution of appropriate substituents on the leaving group or/and acridinium moiety may weaken the bond responsible for the detachment of the leaving group and more efficient transformation into the energy-rich cyclic intermediate (Scheme 1). Investigations on the effect of different substitution sites have already been carried out. These studies focused on substitutions in acridine moiety, especially in positions 2 and/or 7 (Scheme 1, R_2,_ and R_7_, respectively) and 3 and/or 6 (Scheme 1, R_3,_ and R_6_, respectively) [6,38], as well as substitutions in phenyl ring (Scheme 1, R_Ph_) [4,5,6,8] and an endocyclic nitrogen atom (Scheme 1, R_1_) [39,40]. Batmanghelich et al. [39] showed that the chemiluminescence efficiency is 15–20% higher in the case of N-methylacridinium derivative among five studied compounds (R_1_ = CH_3_, C_2_H_5_, *n*C_3_H_7_, *i*C_3_H_7_, and CH_2_Ph), in which the substituent (R_1_) was located at the endocyclic nitrogen atom. Our previous studies [6,7] showed that the derivatives substituted with the methoxy group in acridine moiety (especially in position 2 (Scheme 1, R_2_)) are more effective than unsubstituted derivatives. The dependence is particularly pronounced in aprotic solvents (such as DMSO or acetonitrile), while slightly less in lower alcohols, and in the case of an aqueous media is the opposite [41,42]. In addition, the investigations on the effect of substituents on phenyl moiety of acridinium esters showed that the derivative substituted in the phenyl ring in ortho positions with the electron-donor groups and in the para position with the electron-acceptor group (Scheme 1, R_Ph_) has more than four times higher emission compared with the unsubstituted derivative [43].

To the best of our knowledge, no detailed studies on chemiluminogens with a similar structure to acridinium esters have been carried out so far. However, considering the promising computational results on acridinium thioesters [6,36], it seems interesting to investigate how the change of the leaving group of acridinium ester (phenyl group) affects the efficiency of the chemiluminescence reaction. In this paper, we present the computational results of our studies on the effect of the type of leaving group on the thermodynamic and kinetic parameters of the chemiluminescence reaction. In this work, we focus on comparing the results of quantum-chemical calculations of new potential chemiluminogens with the same values for acridinium esters used so far in immunodiagnostics. The promising results will show us new derivatives or derivatives that could be used as chemiluminogenic fragments in chemiluminescent labels.

## 2. Results and Discussion

### 2.1. Selection of Investigated Objects

Considering our previous studies on the chemiluminescence reaction of acridinium derivatives, e.g., acridinium esters [4,5,7] or acridinium thioesters [6,36], it can be indicated that the type of leaving group is important for the efficiency of the chemiluminescence process. Comparing the energy profiles of the chemiluminescence reaction of acridinium derivatives determined by computational methods [6], it can be seen that the use of a less electronegative atom, that extends the bonds within the leaving group, results in a more efficient transformation of acridinium derivatives into electronically excited products of the CL reaction. Therefore, by making little structural changes (replacing only one atom) in the known and used acridinium esters, it is possible to obtain significant changes in the quantum chemiluminescence efficiency of these compounds. Former studies on acridinium esters revealed, the type of the leaving group primarily determines the kinetics and chemical stability and—to some extent—the efficiency of the emission [4,5,7,11]. The choice of atoms to replace the oxygen atom in the case of acridinium ester [4,5,7] or sulfur atom in the case of acridinium thioester [6,36] was based on three parameters: (i) the electronegativity according to the Pauling scale of the chemical element (R), (ii) the bond length formed between the leaving group and the luminogenic part of the chemiluminogen (C–R), and (iii) ease of synthesis of chemiluminogens (especially a commercial availability of substrates for synthesis). The list of analyzed parameters is presented in Table 1.

As we suggested in our previous publication and confirmed with the results of quantum-chemical calculations [6], the reduction of the electronegativity of the central atom in the leaving group (R) combined with increasing the length of the bond between the leaving group and the luminogenic fragment (C–R) affects easier transformation into electronically excited products of CL reaction. Designing new chemiluminogens, the availability of substrates for future synthesis, and the ease of synthesis of the designed compounds should also be taken into account. In our consideration, we focused on a procedure similar to the already-known synthesis of acridinium esters [4,5]. This synthesis consists of the following three steps: first, acridine-9-carboxylic acid is converted into 9-chlorocarbonylacridine hydrochloride, which is then esterified to form acridine R-ester, which in the final stage is methylated at the endocyclic nitrogen atom to obtain acridinium R-ester. Therefore, the most important seems to be the commercial availability of a suitable R-phenol derivative, which will react with 9-chlorocarbonyl acridine hydrochloride in the second step of the synthesis. From all compounds selected for the study (Table 1), in the case of such derivatives as As-phenol, Si-phenol, or Ge-phenol, despite the general availability of R-phenol derivative, the reaction of this derivative with chlorocarbonyl fragment of organic compound is unknown. Therefore, a method for the synthesis of the desired compound (R-phenyl derivative) should be developed at the beginning. The difficulty of the synthesis is the fundamental problem that can be significant for the exclusion of these derivatives from further studies.

### 2.2. Susceptibility to Nucleophilic Attack

According to our previous studies [4,5,6], acridinium derivatives are sensitive to oxidation in an alkaline environment. In this media, the oxidant, e.g., H_2_O_2_, exists in the anionic form (OOH^−^) and is a nucleophile that reacts with the electrophilic center of acridinium cation. The frontier molecular orbital theory [45] can be helpful in understanding on which atom/group of atoms the electrophilic center of the cation is located. For this reason, the LCAO coefficients of the p_Z_ LUMO orbital were calculated for two potential electrophilic center—the endocyclic carbon atom C9 and the carbonyl carbon atom C15 (Table 1) adjacent to the leaving group. The LCAO coefficients of the p_Z_ LUMO orbital for these two atoms are summarized in Table 2, while Figure S1 in the Supplementary Materials shows the HOMO and LUMO orbitals of all studied acridinium cations. Additionally, Table 2 presents the values of the HOMO–LUMO gap for all considered potential chemiluminogens, according to a rule of thumb that the larger a compound’s HOMO–LUMO gap, the more stable the compound. For instance, a molecule with a high HOMO–LUMO energy gap has low chemical reactivity and high kinetic stability, because it is energetically unfavorable to add an electron to the high-lying LUMO in order to remove electrons from the low-lying HOMO [46,47,48].

The comparison of HOMO and LUMO orbitals (Figure S1 in the Supplementary Materials) shows that the R-phenyl fragment (leaving group) is electron-donating, while acridine moiety (luminescence fragment) is electron-withdrawing. To determine where the electrophilic center of the acridinium cation is located, the LCAO coefficients of the LUMO orbital could be helpful. The LCAO coefficients of the p_Z_ LUMO orbital (Table 2) of two carbon atoms: C9 (carbon atom of acridine moiety to which the carboxyl fragment is attached) and C15 (carbonyl carbon), which may be the most susceptible to the nucleophilic attack by hydroperoxide ions, were compared. Table 2 shows that the LCAO coefficient is many times higher for the carbon atom of acridine moiety (C9) than for the carbonyl carbon atom (C15) (from 11 to 3200 times higher in the case of the S-phenyl and NH-phenyl derivatives, respectively). These results clearly present that the nucleophilic attack of OOH^−^ ions, which starts the chemiluminescence reaction, will occur on the endocyclic carbon atom.

The comparison of the HOMO–LUMO energy gap values (Table 2) shows that all studied compounds are characterized by similar stability (the HOMO–LUMO gap between 2.14 and 2.78 eV for NH-phenyl and Se-phenyl derivatives, respectively). Based on the HOMO–LUMO gap values, none of the proposed compounds can be excluded from further studies. However, the results presented in Table 1 and Table 2 (especially the commercial availability of substrates for synthesis and the HOMO–LUMO gap) show that the Se-phenyl derivative may have synthetic and application potential.

### 2.3. Light Pathway of Chemiluminescence Reaction and Competitive Pathways

**Scheme 2:**
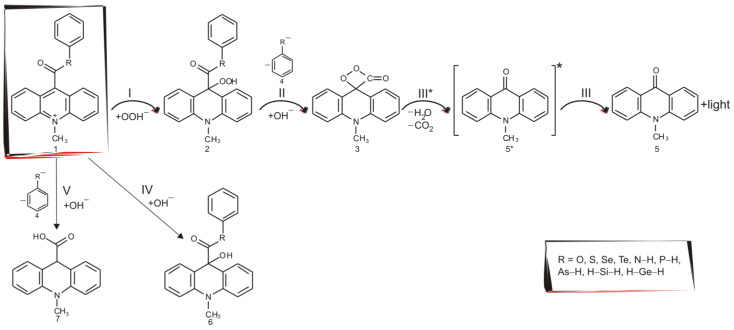
The reaction of acridinium derivatives with hydrogen peroxide anions in an alkaline medium leading to the light emission (steps I, II, III* and III) and the competitive products (step IV leading to the formation of ‘pseudobase’ and step V leading to the hydrolysis of the acridinium cation).

In Scheme 2, based on our previous computational studies [4,5,6], we have presented the chemiluminescence reaction pathway for acridinium derivatives (step I–III) and the formation of competitive reaction products that do not lead to light emission: step IV—reaction with hydroxyl ions and, as a result, the formation of the so-called ‘pseudobase’ (structure **6**) and step V—hydrolysis of acridinium derivative, leading to the formation of 10-methyl-9-carboxylacridinium acid (structure **7**). Table 3 summarizes the results of quantum-chemical calculations of all investigated chemiluminescence reaction steps and competitive reactions for all studied derivatives (Table 1). Additionally, the density functional theory (DFT) optimized geometries of all molecules considered as potential chemiluminogens (lowest energy structures) are shown in Table S2 in the Supplementary Materials.

Based on our previous publications on the mechanism of chemiluminescence of acridinium derivatives—acridinium esters [4,5] and acridinium thioesters [6,36], we proposed a simple mechanism leading to the light emission for studied derivatives, consisting of three steps (Scheme 2):

1. step I—nucleophilic attack of anionic form of oxidant (e.g., OOH^−^) at the C9 atom on acridinium moiety and formation of molecule **2**;
2. step II—reaction of the addition product **2** with hydroxide ions to form cyclic intermediate **3** after elimination of R-phenyl anion (molecule **4**);
3. steps III* and III—a unimolecular decomposition of the cyclic entity **3** after elimination of carbon dioxide and formation of the electronically excited 10-methyl-9-acridinone (molecule 5*), and then returned to the ground state (molecule **5**), by applying the approach:
  - the thermally accessible dioxetanone to reach an electronically excited state of 10-methyl-9-acridinone molecule in S_0_;
  - nonadiabatic transition through spin-orbit coupling between S_0_ and S_1_;
  - the final decomposition to reach 10-methyl-9-acridinone in S_1_.

Additionally, two competitive reactions to the chemiluminescence reaction (dark reaction pathways) should also be considered:

4. step IV—nucleophilic attack of hydroxide ions at the C9 atom on acridinium moiety and formation of so-called pseudobase (molecule **6**);
5. step V—nucleophilic attack of hydroxide ions at the C15 atom of the carbonyl group and formation of 10-methyl-9-carboxyacridinium acid (molecule **7**).

The thermodynamic data summarized in Table 3 for all of the chemiluminescence reaction steps mentioned above show that each step is thermodynamically possible in both the gaseous and aqueous phases. The values of free energy in the aqueous phase are higher than in the gaseous phase, however, the processes are still exothermic (Table 3). In addition, it should be noted that the values of enthalpy and free energy for each step and the most of studied derivatives are similar. The only exceptions are derivatives containing nitrogen and germanium atom in the leaving group (NH-phenyl and GeH_2_-phenyl derivatives, respectively), which in the case of steps II and V have significantly higher values of enthalpy and free energy in gaseous and aqueous phases. These may be due to the leaving group formed in the reaction, i.e., NH-phenyl and GeH_2_-phenyl anions. According to chemical knowledge, nitrogen and germanium atom will not prefer the formation of anionic forms of the compound. Therefore, it will not be thermodynamically stable in the form proposed in the mechanism.

It is also worth noting that in the case of a derivative containing the Te-phenyl group, the values of free energy in the aqueous phase for steps I, IV, and V are significantly lower than in the case of other derivatives. We considered that it might be related to a different basis set for the tellurium atom used in the computational calculation. For this reason, we performed another calculation for two previously studied derivatives (containing oxygen (acridinium ester) and sulfur (acridinium thioester) atoms in leaving group) and one promising derivative (containing selenium atom in leaving group) using the same calculation approach as in the case of derivative containing tellurium atom (DFT method (B3LYP functional) with LanL2DZ (for oxygen, sulfur, and selenium atom) and 6-31G(d,p) (for other atoms) basis sets). The calculation results obtained in this way are summarized in Table S1 in Supplementary Materials. The enthalpy and free enthalpy results for step I calculated using the LanL2DZ basis are not significantly different from those obtained using the 6-31G(d,p) basis set (the values of free energy in aqueous phase calculated with LanL2DZ and 6-31G(d,p) for O–, S– and Se–analogs are −54.2 and −51.5, −42.5 and −43.1, −42.3 and −43.8 kcal mol^−1^, respectively). However, there are some differences in the values of enthalpy and free energy in the gaseous and aqueous phases for steps II (the values of free energy in aqueous phase calculated with LanL2DZ and 6-31G(d,p) for O– and S–analogs are −59.6 and −47.6, −84.0 and −69.0 kcal mol^−1^, respectively), IV (the values of free energy in aqueous phase calculated with LanL2DZ and 6-31G(d,p) for Se–analogs are −62.8 and −43.8 kcal mol^−1^, respectively), and V (the values of free energy in aqueous phase calculated with LanL2DZ and 6-31G(d,p) for O–, S– and Se–analogs are −70.6 and −49.1, −83.3 and −68.9, −86.2 and −60.8 kcal mol^−1^, respectively) within the range of 12–25 kcal mol^−1^. Therefore, we believe that the basis set used slightly affects the obtained values, but it is not a significant difference.

Table 3 also shows the kinetic characteristics of the chemiluminescence reaction. Step III, i.e., the decomposition of cyclic intermediate **3** and subsequent formation of electronically excited reaction product—10-methyl-9-acridinone, is a rate-determining step of the chemiluminescence. For all studied derivatives, this step will proceed in the same way and will have an activation barrier of approximately 16 kcal mol^−1^. In addition, it should be emphasized that steps I and II proceed with a small activation barrier. As we showed in our previous paper [36], these activation barriers are small and do not exceed 5 kcal mol^−1^.

**Figure 1:**
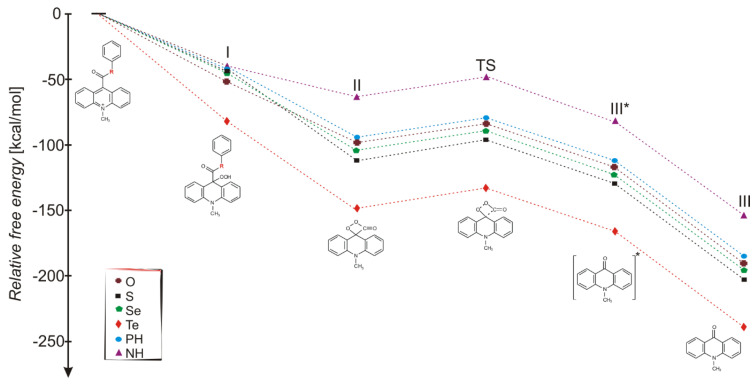
The diagram of relative free energy in the aqueous phase of the reaction leading to the light emission (step I, II, III* and III, Scheme 2) of the selected acridinium R-esters (Table 1).

In order to more accurately compare the thermodynamic characteristics of the studied derivatives, the free enthalpy profiles in the aqueous phase for all steps of the chemiluminescence reaction were plotted (Figure 1). Only derivatives that can be easily synthesized (discussed in the Section 2.1) were taken into account. After analyzing these free energy profiles of the whole reaction leading to light generation, one thing that can be noticed is that the first two steps determine the thermodynamic predominance of the process. From Figure 1 can be seen that compared to acridinium esters, which are well known and used in immunological diagnostics, better thermodynamic characteristics after the first two steps of the chemiluminescence reaction have derivatives containing sulfur, selenium, and tellurium atom in leaving group. As against acridinium ester, the values of free enthalpy in the aqueous phase after step II are lower by 5.5, 13.0, and 49.5 kcal mol^−1^ in the case of derivatives containing selenium, sulfur, and tellurium, respectively. These results indicate that each of these derivatives can be an efficient chemiluminogen and find application in medical or chemical analysis.

In order to consider the utilitarian perspectives of the studied chemiluminogens (derivative containing sulfur, selenium, tellurium, nitrogen, and phosphorus in leaving group) and currently used in the analysis—acridinium ester, the free energy values of step I (step starting the light pathway), step IV (leading to the formation of pseudobase) and step V (leading to the hydrolysis of acridinium derivative) were compared. Figure S2 in the Supplementary Materials presents the free energy profiles in the aqueous phase of these three steps for all derivatives mentioned above. The computational results show that steps initiating the dark pathways, which are not leading to light generation (formation of ‘pseudobase’ and hydrolysis of the compound), are thermodynamically preferable. The differences between the values of free enthalpy in the aqueous phase for step, I and step IV are similar and amount to about 20 kcal mol^−1^ in favor of the formation of ‘pseudobase’. For step V leading to the hydrolysis of acridinium cation, in the case of the compound containing oxygen and phosphorus in leaving group, the values of free enthalpy in the aqueous phase are higher in the case of the reaction leading to the hydrolysis of the compound (by 2.4 and 21.5 kcal mol^−1^, respectively) than the reaction leading to light generation. For other derivatives, the hydrolysis reaction is thermodynamically preferred (from 9.7 for the nitrogen-containing derivative to 25.8 kcal mol^−1^ for the sulfur-containing derivative). These show how important the selection of measurement conditions will be to eliminate the contribution of undesirable reactions on the efficiency of the chemiluminescence process.

An interesting aspect of the newly designed chemiluminogens will also be the pH range of chemiluminescence measurements. According to the literature [4,5], it is known that acridinium ester achieves the most efficient emission at the solution with pH~12. The addition of electron-withdrawing substituents in the phenyl ring reduces the values of pH of CL measurements [4,5,11]. This is related to the weakening of the C–O bond and the easier detachment of the leaving group [4,5]. This is also visible in the LCAO coefficients of the p_Z_ LUMO orbital—the higher the electron deficit at C9 carbon, the easier the chemiluminescence reaction occurs [4,5,6]. Therefore, it can be expected that replacing oxygen atoms with atoms such as sulfur, selenium or tellurium will shift the pH value of the measurements towards lower values (closer to a pH value of 7).

## 3. Materials and Methods

The density functional theory (DFT) [49] with Becke’s three-parameter hybrid method with LYP functional (B3LYP) [50,51] was used to calculate the equilibrium geometries of all structures shown in Scheme 2. The LanL2DZ [52,53] and the 6-31G(d,p) [54,55] basis sets were employed for the tellurium atom and other atoms, respectively. Selecting the method and the type of function to study the chemiluminescence reaction of a new group of chemiluminogens, we considered our [4,5,6,7,36] and other authors [56] investigations, which show that the chosen method could produce reliable results for the thermodynamic data and provide accurate qualitative results for the mechanism of chemiluminescence reaction of investigated molecules. Next, harmonic vibrational frequencies were computed (at the same level of theory) and all obtained structures have been identified as minima with all positive frequencies (reactants, product complexes, and products) or transition states having only one imaginary frequency on the potential energy surface. Intrinsic reaction coordinate (IRC) [57] calculations were also performed at the same level of theory to obtain the minimum energy path. The geometries and frequency calculation of the ground state were calculated with an open-shell (U) approach for transition states and a close-shell (R) approach for reactants/products. The broken-symmetry (BS) technology is adopted in unrestricted open-shell calculations. For the solvent effect, the polarizable continuum model (PCM) was utilized [58], which mimics the solvent environment (UAHF radii were used to obtain the molecular cavity) [59] and assumes a complete uniformity of the medium [60]. The vertical transition energies for the electronically excited 10-methyl-9-acridinone were computed from an excited state-optimized structure using the state-specific approach in solution using the time-dependent DFT [61] method with the B3LYP functional [50,51] and the 6-31G(d,p) [54,55] basis set. All calculations were performed using the Gaussian16 program package [62]. The ChemCraft software (Version 1.8) [63] was used to visualize the results of the quantum chemical calculations.

## 4. Conclusions

The increase in diseases in the world makes it matters to search for new, effective diagnostic methods that will help diagnose the disease in a quick and simple way. One of the promising immunological diagnostic procedures is chemiluminescent labeling, which requires effective chemiluminogens, such as the currently used acridinium esters. However, despite the many advantages of acridinium esters, and widely used in CL immunoassays, attention is paid to the inherent instability of these compounds. Therefore, it should be considered whether more efficient chemiluminogens could be found among acridinium derivatives.

Initial work on a new group of compounds—potential chemiluminogens—began with modeling derivatives structurally similar, but differing in one atom from acridinium esters—replacing the oxygen atom in the sensitive place of leaving the group with other atoms/groups such as sulfur, selenium, tellurium, phosphorus (P–H), nitrogen (N–H), arsenic (As–H), silicon (H–Si–H), germanium (H–Ge–H). Next, we studied the thermodynamic and kinetic profiles of the mechanism of chemiluminescence reaction and competitive reactions (formation of ‘pseudobase’ and hydrolysis of the compound). Based on the computational results and comparing the energy profiles of the chemiluminescence reaction of potential chemiluminogens and acridinium ester can be seen that derivatives containing sulfur, selenium, and tellurium have a more favorable thermodynamic and kinetic profile of reaction leading to light generation. In addition, it should be noted that all of the mentioned above derivatives similar to acridinium ester willingly undergo the reaction leading to the formation of ‘pseudobase’. All of the studied derivatives are also susceptible to hydrolysis of the compound. However, it should be noted that the compound containing selenium will be the most stable, followed by the compound containing tellurium and sulfur. The factors determining the choice of the derivative for further experimental studies should also include the ease of synthesis of potential chemiluminogens and the availability of the necessary substrates. Therefore, chemiluminogens containing selenium, sulfur, and tellurium seem to be the most promising.

To sum up, with the use of a simple computational approach, we were able to identify potential chemiluminogens (containing selenium, sulfur, and tellurium instead of oxygen in acridinium ester) for further experimental studies. Synthesis, followed by studies of their chemiluminescent properties, and ultimately in chemiluminescent labeling, are further steps to confirm their potential applicability in immunodiagnostics.

## Figures and Tables

**Table 1:** Objects considered for studies using computational chemistry methods.

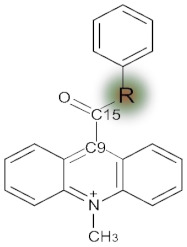	**R**	**Electronegativity (χ) in the Pauling Scale [44]**	**C–R** **Bond Length** **(Å)**	**Ease of Synthesis/Availability of Substrates**
	O	3.44	1.342 [6]	+
	S	2.58	1.794 [6]	+
	Se	2.55	1.923	+
	Te	2.10	2.199	+
	N (N–H)	3.04	1.359	+
	P (P–H)	2.19	1.887	+
	As (As–H)	2.18	2.018	+/−
	Si (H–Si–H)	1.90	1.958	+/−
	Ge (H–Ge–H)	2.01	2.013	+/−

**Table 2:** The value of LCAO coefficients of p_Z_ LUMO orbital at the potential electrophilic center (endocyclic carbon atom C9 and carboxylic carbon atom C15, see Table 1).

R	LCAO Coefficient of p_Z_ LUMO Orbital at		HOMO–LUMO Gap (eV)
	C9	C15	
O	0.3145	0.0257	2.53
S	0.3154	0.0275	2.62
Se	0.3266	0.0098	2.78
Te	0.3405	0.0170	2.23
N (N–H)	0.3265	0.0001	2.14
P (P–H)	0.3121	0.0255	2.40
As (As–H)	0.3333	0.0027	2.44
Si (H–Si–H)	0.3180	0.0197	2.73
Ge (H–Ge–H)	0.3387	0.0030	2.63

**Table 3:** The thermodynamic and kinetic data of the light pathway of chemiluminescence reaction (step I, II, TS, III* and III, Scheme 2) and the competitive pathways (step IV—formation of the ‘pseudobase’ and step V—hydrolysis of the acridinium cation, Scheme 2).

Thermodynamic Characteristic									
Step No. (Scheme 2)	R	Gaseous Phase		Aqueous Phase	Step No. (Scheme 2)	R	Gaseous Phase		Aqueous Phase
		Δ_r,298_*H*^0^	Δ_r,298_*G*^0^	Δ_r,298_*G*^0^			Δ_r,298_*H*^0^	Δ_r,298_*G*^0^	Δ_r,298_*G*^0^
I	O	−166.6 *^a^*	−153.6 *^a^*	−51.5 *^a^*	II	O	−60.5 *^b^*	−73.7 *^b^*	−47.6 *^b^*
	S	−166.2 *^b^*	−153.0 *^b^*	−43.1 *^b^*		S	−80.1 *^b^*	−93.2 *^b^*	−69.0 *^b^*
	Se	−166.3	−153.1	−43.8		Se	−73.1	−86.4	−60.8
	Te	−165.8	−198.8	−82.7		Te	−104.1	−117.1	−65.9
	N (N–H)	−167.1	−153.7	−42.6		N (N–H)	−34.7	−48.8	−21.7
	P (P–H)	−166.4	−153.1	−42.8		P (P–H)	−66.8	−79.9	−52.9
	As (As–H)	−163.9	−150.5	−42.6		As (As–H)	−64.6	−77.1	−48.1
	Si (H–Si–H)	−167.3	−153.8	−36.3		Si (H–Si–H)	−55.4	−69.0	−49.4
	Ge (H–Ge–H)	−168.4	−155.4	−45.8		Ge (H–Ge–H)	−30.6	−42.8	−14.0
III*		−15.5 *^b^*	−27.2 *^b^*	−33.2 *^b^*	III		−78.1 *^b^*	−77.6 *^b^*	−73.3 *^b^*
IV	O	−186.1 *^a^*	−175.6 *^a^*	−73.3 *^a^*	V	O	−72.5 *^b^*	−74.3 *^b^*	−49.1 *^b^*
	S	−197.1 *^b^*	−187.5 *^b^*	−61.9 *^b^*		S	−92.0 *^b^*	−93.2 *^b^*	−68.9 *^b^*
	Se	−192.0	−181.0	−63.5		Se	−85.0	−86.5	−61.4
	Te	−191.5	−179.2	−103.2		Te	−115.5	−162.9	−105.4
	N (N–H)	−194.3	−182.3	−63.4		N (N–H)	−47.5	−49.5	−21.1
	P (P–H)	−192.5	−181.3	−63.4		P (P–H)	−78.9	−80.0	−52.5
	As (As–H)	−195.1	−183.0	−64.9		As (As–H)	−74.1	−74.7	−47.5
	Si (H–Si–H)	−194.0	−182.3	−64.6		Si (H–Si–H)	−68.4	−69.8	−42.5
	Ge (H–Ge–H)	−194.6	−183.0	−66.5		Ge (H–Ge–H)	−44.7	−45.2	−16.6
**Kinetic Characteristic**									
**Step No.** **(Scheme 2)**	**Gaseous Phase**							**Aqueous Phase**	
	**Δ_a,298_*H*^0^**		**Δ_a,298_*G*^0^**	**_298_*k*^0^ (_298_*τ*_99_)**				**Δ_a,298_*G*^0^**	
TS	12.9 *^b^*		14.2 *^b^*	2.5 × 10^2^ (1.9 × 10^−2^) *^b^*				15.6 *^b^*	

Δ_r,298_*H*^0^ and Δ_r,298_*G*^0^ (both in kcal mol^−1^), respectively, represent the enthalpy and Gibbs’ free energy (gaseous phase) or free energy (aqueous phase) of the reaction corresponding to a given step number at standard temperature and pressure; Δ_a,298_*H*^0^ and Δ_a,298_*G*^0^ (both in kcal mol^−1^), respectively, represent the enthalpy and Gibbs’ free energy (gaseous phase) or free energy (aqueous phase) of activation at standard temperature and pressure; _298_*k*^0^ (in s^−1^) and _298_*τ*_99_, respectively, denote the rate constant and the time after which the reaction is 99% complete; *^a^* [4]; *^b^* [6].

## Data Availability

Not applicable.

## Supplementary Materials

The supporting information can be downloaded at: https://www.mdpi.com/article/10.3390/ijms24119468/s1.

## Abbreviations

CL	Chemiluminescence
DFT	Density functional theory
HOMO	Highest Occupied Molecular Orbital
LCAO	Linear combination of atomic orbitals
LUMO	Lowest Unoccupied Molecular Orbital
PCM	Polarizable Continuum Model
TD DFT	Time dependent density functional theory
TS	Transition State
