# Supplementary material for: What Other Than Acridinium Esters? Computational Search for New Acridinium-Based Chemiluminogens
Source: Int J Mol Sci. 2023 May 30;24(11):9468. doi: 10.3390/ijms24119468 (PMC10253539; doi:10.3390/ijms24119468)

# Supplementary Materials

# What else than acridinium esters?
# Computational search for new acridinium-based chemiluminogens

**Milena Pieńkos and Beata Zadykowicz***

*Faculty of Chemistry, University of Gdańsk,*
*Wita Stwosza 63, 80-308 Gdańsk, Poland*

**Correspondence: beata.zadykowicz@ug.edu.pl*

**Figure S1.** The Highest Occupied Molecular Orbital (HOMO) and Lowest Unoccupied Molecular Orbital (LUMO) of investigated potential chemiluminogens (R – the central atom in leaving group).

| R | orbital | | R | orbital | | R | orbital | |
|---|---|---|---|---|---|---|---|---|
| | HOMO | LUMO | | HOMO | LUMO | | HOMO | LUMO |
| **O** | 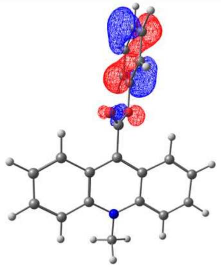 | 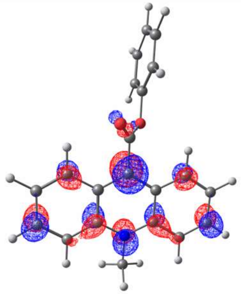 | **S** | 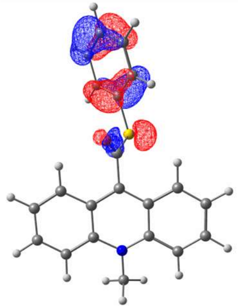 | 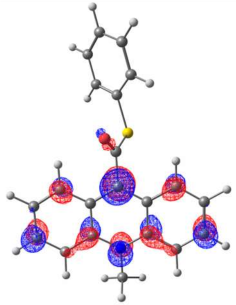 | **Se** | 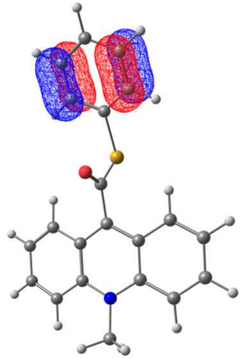 | 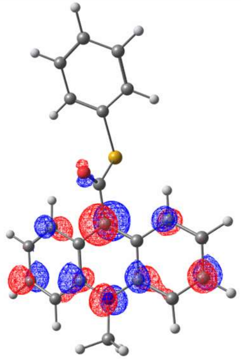 |
| **Te** | 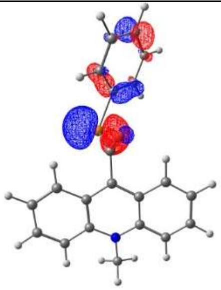 | 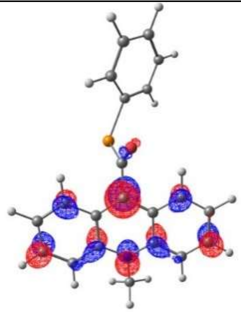 | **N (N–H)** | 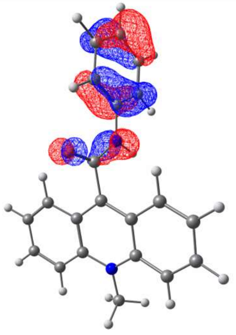 | 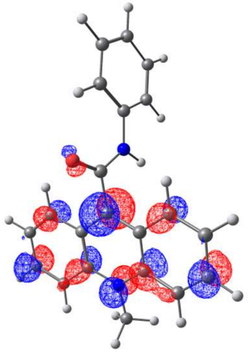 | **P (P–H)** | 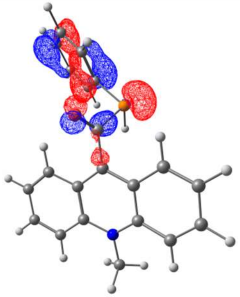 | 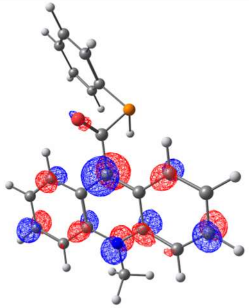 |
| **As (As–H)** | 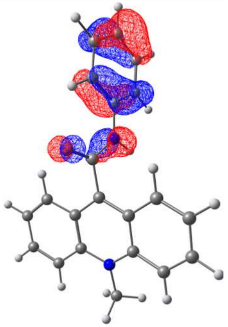 | 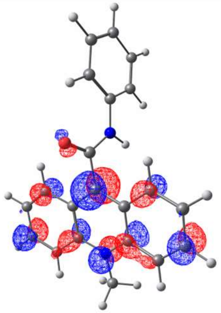 | **Si (H–Si–H)** | 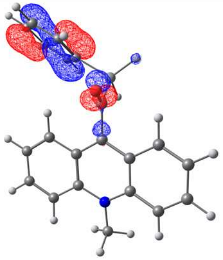 | 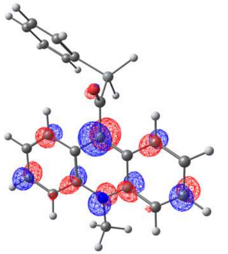 | **Ge (H–Ge–H)** | 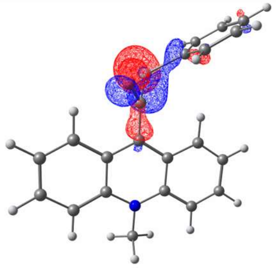 | 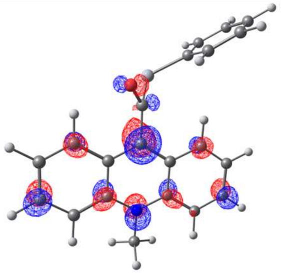 |

**Table S1.** The thermodynamic data of the light pathway of chemiluminescence reaction (step I, II, Scheme 1) and the competitive pathways (step IV – formation of the 'pseudobase' and step V – hydrolysis of the acridinium cation, Scheme 1) calculated with different basis sets (LanL2DZ and 6-31G(d,p)) for selected derivatives (R).

| Step no. (Scheme 1) | R | Basis set for R | gaseous phase | | aqueous phase | Step no. (Scheme 1) | R | Basis set for R | gaseous phase | | aqueous phase |
|---|---|---|---|---|---|---|---|---|---|---|---|
| | | | $\Delta_{r,298}H^0$ | $\Delta_{r,298}G^0$ | $\Delta_{r,298}G^0$ | | | | $\Delta_{r,298}H^0$ | $\Delta_{r,298}G^0$ | $\Delta_{r,298}G^0$ |
| **I** | Te | LanL2DZ | −165.8 | −198.8 | −82.7 | II | Te | LanL2DZ | −104.1 | −117.1 | −65.9 |
| | O | 6-31G(d,p) | −166.6 [a] | −153.6 [a] | −51.5 [a] | | O | 6-31G(d,p) | −60.5 | −73.7 | −47.6 |
| | | LanL2DZ | −175.1 | −162.3 | −54.2 | | | LanL2DZ | −71.1 | −85.1 | −59.6 |
| | S | 6-31G(d,p) | −166.2 [b] | −153.0 [b] | −43.1 [b] | | S | 6-31G(d,p) | −80.1 [b] | −93.2 [b] | −69.0 [b] |
| | | LanL2DZ | −165.1 | −152.1 | −42.5 | | | LanL2DZ | −96.2 | −108.8 | −84.0 |
| | Se | 6-31G(d,p) | −166.3 | −153.1 | −43.8 | | Se | 6-31G(d,p) | −73.1 | −86.4 | −60.8 |
| | | LanL2DZ | −165.2 | −153.6 | −42.3 | | | LanL2DZ | −94.6 | −103.9 | −62.8 |
| **IV** | Te | LanL2DZ | −191.5 | −179.2 | −103.2 | V | Te | LanL2DZ | −115.5 | −162.9 | −105.4 |
| | O | 6-31G(d,p) | −186.1 [a] | −175.6 [a] | −73.3 [a] | | O | 6-31G(d,p) | −72.5 | −74.3 | −49.1 |
| | | LanL2DZ | −196.2 | −186.6 | −70.1 | | | LanL2DZ | −92.9 | −94.4 | −70.6 |
| | S | 6-31G(d,p) | −197.1 [b] | −187.5 [b] | −61.9 [b] | | S | 6-31G(d,p) | −92.0 [b] | −93.2 [b] | −68.9 [b] |
| | | LanL2DZ | −188.9 | −187.0 | −60.9 | | | LanL2DZ | −107.0 | −107.9 | −83.3 |
| | Se | 6-31G(d,p) | −166.3 | −153.1 | −43.8 | | Se | 6-31G(d,p) | −73.1 | −86.4 | −60.8 |
| | | LanL2DZ | −191.2 | −182.7 | −62.8 | | | LanL2DZ | −109.1 | −111.5 | −86.2 |

$\Delta_{r,298}H^0$ and $\Delta_{r,298}G^0$ (both in kcal mol$^{-1}$), respectively, represent the enthalpy and Gibbs' free energy (gaseous phase) or free energy (aqueous phase) of the reaction corresponding to a given step number at standard temperature and pressure;
[a] [1]; [b] [2].


[1] Krzymiński, K.; Ożóg, A.; Malecha, P.; Roshal, A.D.; Wróblewska, A.; Zadykowicz, B.; Błażejowski, J. Chemiluminogenic Features of 10-Methyl-9-(Phenoxycarbonyl)Acridinium Trifluoromethanesulfonates Alkyl Substituted at the Benzene Ring in Aqueous Media. *J. Org. Chem.* **2011**, *76*, 1072–1085. https://doi.org/10.1021/jo1020882.

[2] Pieńkos, M.; Zadykowicz, B. Computational Insights on the Mechanism of the Chemiluminescence Reaction of New Group of Chemiluminogens – 10-Methyl-9-thiophenoxycarbonylacridinium Cations. *Int. J. Mol. Sci.* **2020**, *21*, 4417. https://doi.org/10.3390/ijms21124417.

**Figure S2.** The diagram of relative free energy in aqueous phase of the first steps of reaction leading to the light emission (step I, Scheme 1) and competitive reactions (step IV – formation of 'pseudobase' and step V – formation of 10-methyl-9-carboxyacridinium acid (hydrolysis of the compound)) of the selected acridinium R-esters (Table 1).

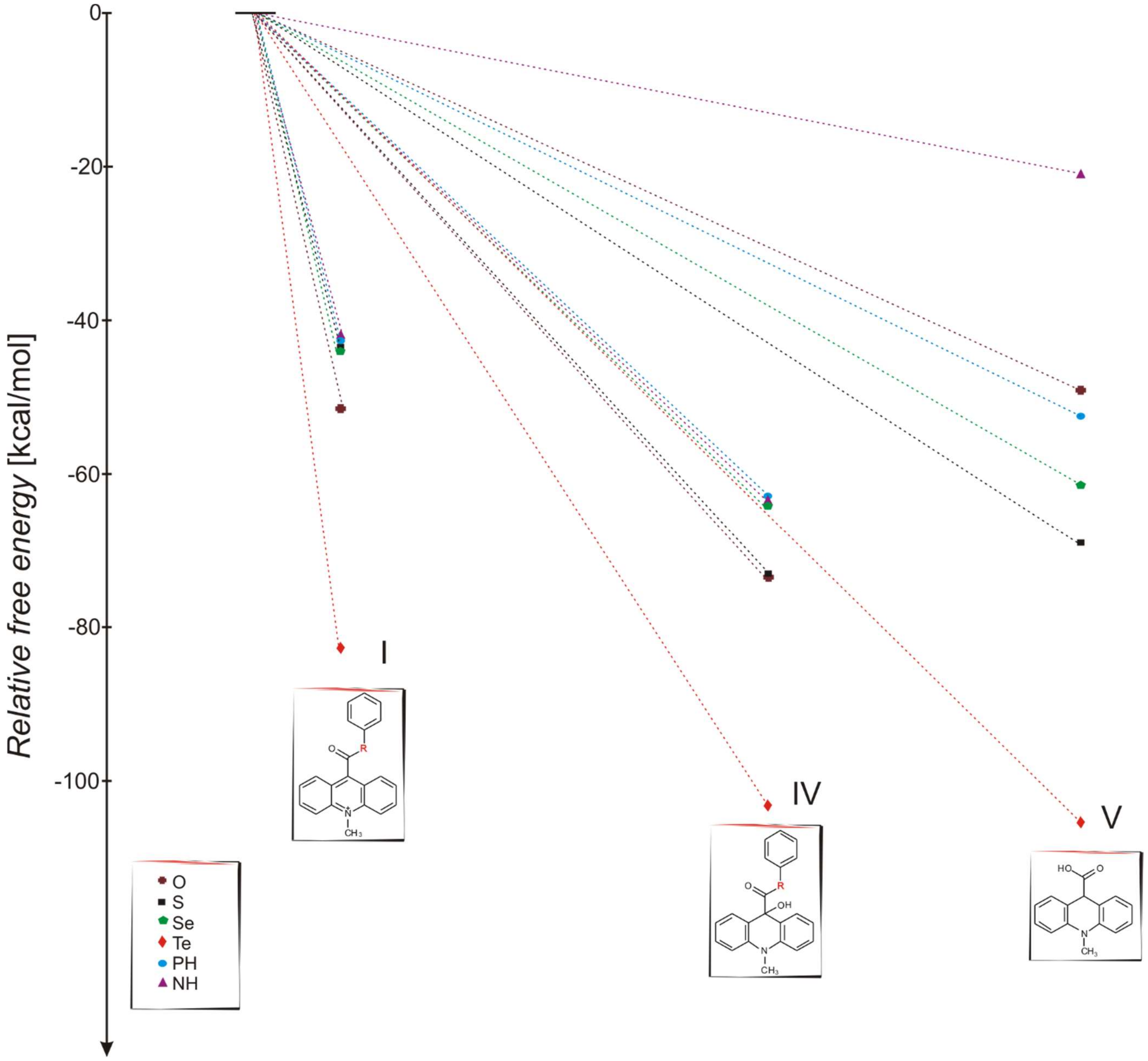

**Table S2.** Cartesian coordinates and structures of the lowest energy structures of investigated molecules. List of abbreviations: * – excited product, $S_1$ – first singlet state.

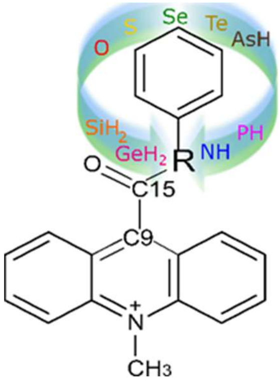


| **R = Se** | | | |
|---|---|---|---|
| **Structure no. 1**<br>*E* = –3339.05392109 Ha | | | |
| Atom | X | Y | Z |
| C | 3.026493000 | 1.205623000 | 0.132682000 |
| C | 3.718741000 | 2.438874000 | 0.067188000 |
| C | 3.014903000 | 3.620651000 | -0.037465000 |
| C | 1.600712000 | 3.642912000 | -0.091613000 |
| C | 0.904764000 | 2.464152000 | -0.037858000 |
| C | 1.590978000 | 1.219195000 | 0.084490000 |
| H | 4.799042000 | 2.472951000 | 0.058936000 |
| H | 3.565464000 | 4.553752000 | -0.100840000 |
| H | 1.077864000 | 4.586895000 | -0.196166000 |
| H | -0.173409000 | 2.442833000 | -0.144760000 |
| C | 0.896503000 | -0.000046000 | 0.146445000 |
| C | 1.591220000 | -1.219134000 | 0.084562000 |
| C | 3.026745000 | -1.205252000 | 0.132718000 |
| N | 3.688225000 | 0.000254000 | 0.251494000 |
| C | -0.595391000 | -0.000038000 | 0.426849000 |
| C | 5.142636000 | 0.000343000 | 0.513242000 |
| C | 0.905271000 | -2.464256000 | -0.037690000 |
| C | 1.601474000 | -3.642866000 | -0.091471000 |
| C | 3.015659000 | -3.620288000 | -0.037405000 |
| C | 3.719251000 | -2.438360000 | 0.067231000 |
| H | -0.172903000 | -2.443186000 | -0.144586000 |
| H | 1.078830000 | -4.586966000 | -0.195994000 |
| H | 3.566430000 | -4.553262000 | -0.100846000 |
| H | 4.799559000 | -2.472192000 | 0.058952000 |
| H | 5.400583000 | 0.876162000 | 1.102711000 |
| H | 5.400579000 | -0.875196000 | 1.103142000 |
| H | 5.709299000 | 0.000143000 | -0.421747000 |
| Se | -1.714309000 | -0.000537000 | -1.132899000 |
| O | -0.969538000 | 0.000408000 | 1.575058000 |
| C | -3.423868000 | -0.000150000 | -0.255519000 |
| C | -4.043887000 | 1.216285000 | 0.039046000 |
| C | -5.298761000 | 1.211153000 | 0.647571000 |
| C | -5.923269000 | 0.000248000 | 0.952905000 |
| C | -5.298646000 | -1.210868000 | 0.648609000 |
| C | -4.043804000 | -1.216391000 | 0.040057000 |
| H | -3.558192000 | 2.153953000 | -0.208634000 |
| H | -5.787755000 | 2.151717000 | 0.880376000 |
| H | -6.900928000 | 0.000411000 | 1.424378000 |
| H | -5.787548000 | -2.151287000 | 0.882193000 |
| H | -3.558014000 | -2.154209000 | -0.206901000 |

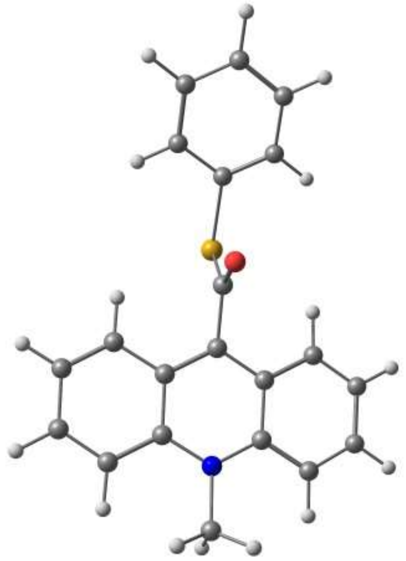

| R = Se | | | |
|---|---|---|---|
| **Structure no. 2** | | | |
| $E$ = –3490.21432860 Ha | | | |
| Atom | X | Y | Z |
| C | 1.454506000 | -2.450311000 | 0.884827000 |
| C | 1.642049000 | -1.134024000 | 0.437317000 |
| C | 2.477137000 | -0.898320000 | -0.678658000 |
| C | 3.153006000 | -1.991721000 | -1.258926000 |
| C | 2.963359000 | -3.283097000 | -0.783534000 |
| C | 2.102922000 | -3.524236000 | 0.287678000 |
| C | 0.951421000 | 0.003732000 | 1.163136000 |
| C | 1.382167000 | 1.366696000 | 0.650542000 |
| C | 2.206698000 | 1.507822000 | -0.485372000 |
| N | 2.634749000 | 0.389700000 | -1.202428000 |
| C | 2.602405000 | 2.807630000 | -0.865058000 |
| C | 2.155662000 | 3.923783000 | -0.168424000 |
| C | 1.312849000 | 3.783729000 | 0.935243000 |
| C | 0.941261000 | 2.506386000 | 1.335504000 |
| C | -0.592927000 | -0.071091000 | 1.014365000 |
| O | -1.371123000 | 0.283209000 | 1.866980000 |
| O | 1.281443000 | 0.070220000 | 2.572143000 |
| O | 0.594091000 | -0.969624000 | 3.322573000 |
| C | 3.230423000 | 0.565038000 | -2.520884000 |
| Se | -1.111786000 | -0.723849000 | -0.746682000 |
| C | -2.970695000 | -0.234969000 | -0.631562000 |
| C | -3.389725000 | 1.023464000 | -1.071449000 |
| C | -4.741076000 | 1.364774000 | -1.011220000 |
| C | -5.671844000 | 0.451179000 | -0.513099000 |
| C | -5.251199000 | -0.805174000 | -0.073750000 |
| C | -3.900568000 | -1.150380000 | -0.132728000 |
| H | 3.284802000 | 2.948298000 | -1.693362000 |
| H | 2.481267000 | 4.909538000 | -0.487907000 |
| H | 0.960658000 | 4.653252000 | 1.480040000 |
| H | 0.299948000 | 2.368078000 | 2.198026000 |
| H | 0.803203000 | -2.612586000 | 1.734129000 |
| H | 1.944216000 | -4.532171000 | 0.656451000 |
| H | 3.499514000 | -4.102811000 | -1.253018000 |
| H | 3.851688000 | -1.831532000 | -2.069949000 |
| H | 2.793191000 | 1.439613000 | -3.003660000 |
| H | 4.320866000 | 0.690224000 | -2.485186000 |
| H | 2.996861000 | -0.299176000 | -3.143258000 |
| H | -3.567360000 | -2.125283000 | 0.207623000 |
| H | -5.972864000 | -1.517044000 | 0.316069000 |
| H | -6.723647000 | 0.717961000 | -0.467766000 |
| H | -5.065321000 | 2.343733000 | -1.351895000 |
| H | -2.661385000 | 1.730438000 | -1.455323000 |
| H | -0.270411000 | -0.533479000 | 3.445918000 |

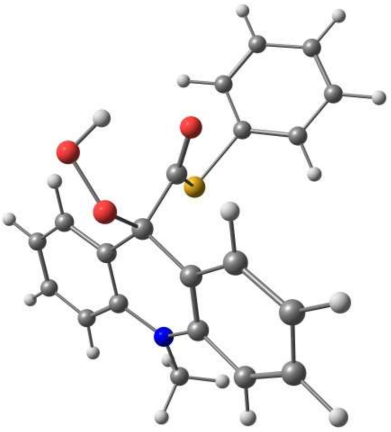

| R = Se | | | |
|---|---|---|---|
| **Structure no. 4** | | | |
| $E$ = –2631.07370596 Ha | | | |
| Atom | X | Y | Z |
| C | 0.000000000 | -0.029477000 | 0.000000000 |
| C | -1.198754000 | -0.787255000 | 0.000000000 |
| C | -1.200325000 | -2.179999000 | 0.000000000 |
| C | 0.000000000 | -2.900719000 | 0.000000000 |
| C | 1.200325000 | -2.179999000 | 0.000000000 |
| C | 1.198754000 | -0.787256000 | 0.000000000 |
| H | -2.140954000 | -0.245081000 | 0.000000000 |
| H | 2.140954000 | -0.245081000 | 0.000000000 |
| Se | 0.000000000 | 1.855686000 | 0.000000000 |
| H | -2.151472000 | -2.713114000 | 0.000000000 |
| H | 0.000000000 | -3.988703000 | 0.000000000 |
| H | 2.151472000 | -2.713114000 | 0.000000000 |

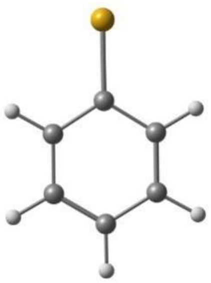

**R = Se**

**Structure no. 6**

*E* = –3415.08976629 Ha

| Atom | X | Y | Z |
|---|---|---|---|
| C | 3.631695000 | -1.211021000 | -0.336270000 |
| C | 2.942199000 | -0.000020000 | -0.436718000 |
| C | 3.631719000 | 1.210988000 | -0.336522000 |
| C | 5.012411000 | 1.209013000 | -0.135834000 |
| C | 5.702874000 | -0.000002000 | -0.035776000 |
| C | 5.012370000 | -1.209030000 | -0.135591000 |
| Se | 1.044003000 | 0.000124000 | -0.759201000 |
| C | 0.490016000 | 0.000156000 | 1.098656000 |
| O | 1.269093000 | 0.000220000 | 2.024594000 |
| C | -1.057390000 | -0.000025000 | 1.374159000 |
| C | -1.675618000 | -1.247725000 | 0.754770000 |
| C | -2.123352000 | -1.210268000 | -0.581335000 |
| N | -2.051452000 | -0.000051000 | -1.295081000 |
| C | -2.123503000 | 1.210118000 | -0.581290000 |
| C | -1.675752000 | 1.247604000 | 0.754811000 |
| C | -1.765980000 | -2.431654000 | 1.484460000 |
| C | -2.269827000 | -3.594967000 | 0.904653000 |
| C | -2.696353000 | -3.563245000 | -0.422586000 |
| C | -2.635547000 | -2.382333000 | -1.160324000 |
| C | -2.635766000 | 2.382166000 | -1.160275000 |
| C | -2.696600000 | 3.563090000 | -0.422561000 |
| C | -2.270037000 | 3.594846000 | 0.904665000 |
| C | -1.766138000 | 2.431556000 | 1.484466000 |
| C | -2.196153000 | -0.000047000 | -2.739897000 |
| H | -3.014150000 | -2.370708000 | -2.175542000 |
| H | -3.098563000 | -4.457838000 | -0.889251000 |
| H | -2.328015000 | -4.512441000 | 1.481445000 |
| H | -1.438581000 | -2.420351000 | 2.518059000 |
| H | -1.438681000 | 2.420291000 | 2.518049000 |
| H | -2.328194000 | 4.512328000 | 1.481447000 |
| H | -3.098866000 | 4.457653000 | -0.889234000 |
| H | -3.014353000 | 2.370492000 | -2.175497000 |
| H | -1.698938000 | -0.882299000 | -3.147515000 |
| H | -3.243525000 | 0.000042000 | -3.075167000 |
| H | -1.698775000 | 0.882112000 | -3.147537000 |
| H | 3.090590000 | 2.148337000 | -0.414389000 |
| H | 5.546609000 | 2.151244000 | -0.056695000 |
| H | 6.777793000 | -0.000028000 | 0.119303000 |
| H | 5.546587000 | -2.151238000 | -0.056286000 |
| H | 3.090504000 | -2.148356000 | -0.413936000 |
| O | -1.206438000 | 0.000011000 | 2.776686000 |
| H | -0.300204000 | -0.000153000 | 3.136915000 |

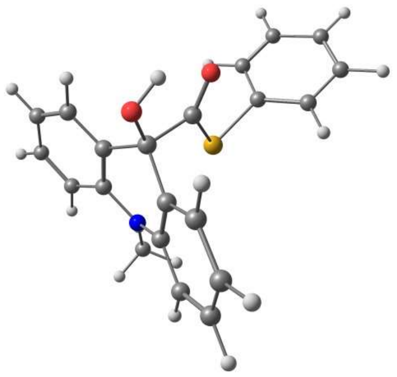

| R = Te | | | |
|---|---|---|---|
| **Structure no. 1** $E$ = –947.677215217 Ha | | | |
| Atom | X | Y | Z |
| C | -4.062906000 | -0.842494000 | 0.983458000 |
| C | -3.383205000 | 0.115720000 | 0.220410000 |
| C | -3.928313000 | 0.588652000 | -0.979511000 |
| C | -5.155338000 | 0.088819000 | -1.418856000 |
| C | -5.839760000 | -0.861856000 | -0.659461000 |
| C | -5.296094000 | -1.323654000 | 0.541018000 |
| Te | -1.516203000 | 0.870465000 | 0.919442000 |
| C | -0.405409000 | 0.003896000 | -0.768783000 |
| O | -0.939575000 | -0.430581000 | -1.758631000 |
| C | 1.081673000 | -0.104461000 | -0.531125000 |
| C | 1.652757000 | -1.384340000 | -0.357479000 |
| C | 3.035789000 | -1.482067000 | 0.021711000 |
| N | 3.767743000 | -0.330291000 | 0.233832000 |
| C | 3.270979000 | 0.914748000 | -0.090432000 |
| C | 1.890731000 | 1.047924000 | -0.471736000 |
| C | 3.618642000 | -2.764407000 | 0.165750000 |
| C | 2.857724000 | -3.897157000 | -0.033505000 |
| C | 1.488223000 | -3.813951000 | -0.376499000 |
| C | 0.900924000 | -2.585291000 | -0.536672000 |
| C | 4.089219000 | 2.069944000 | -0.067151000 |
| C | 3.561879000 | 3.300446000 | -0.400271000 |
| C | 2.206419000 | 3.446444000 | -0.774701000 |
| C | 1.392619000 | 2.342952000 | -0.813689000 |
| C | 5.119354000 | -0.435341000 | 0.821490000 |
| H | 4.669283000 | -2.875753000 | 0.394055000 |
| H | 3.327519000 | -4.870497000 | 0.066615000 |
| H | 0.911389000 | -4.719898000 | -0.526178000 |
| H | -0.138213000 | -2.510542000 | -0.830181000 |
| H | 0.361334000 | 2.437815000 | -1.131035000 |
| H | 1.820792000 | 4.422423000 | -1.047642000 |
| H | 4.211774000 | 4.169770000 | -0.392550000 |
| H | 5.142060000 | 2.001860000 | 0.168156000 |
| H | 5.150540000 | -1.285411000 | 1.498130000 |
| H | 5.879400000 | -0.550307000 | 0.043672000 |
| H | 5.324755000 | 0.455048000 | 1.410117000 |
| H | -3.407854000 | 1.335974000 | -1.568040000 |
| H | -5.578132000 | 0.450134000 | -2.351339000 |
| H | -6.797868000 | -1.240968000 | -1.001575000 |
| H | -5.827604000 | -2.062397000 | 1.133202000 |
| H | -3.639672000 | -1.211906000 | 1.912026000 |

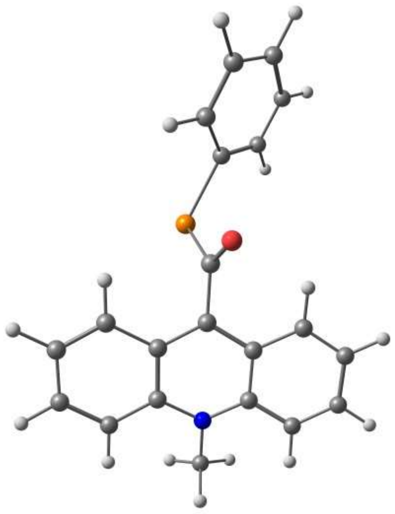

| R = Te | | | |
|---|---|---|---|
| **Structure no. 2**<br>*E* = –1098.83706374 Ha | | | |
| Atom | X | Y | Z |
| C | -3.876335000 | -0.736580000 | 0.549231000 |
| C | -3.112692000 | -0.241706000 | -0.514872000 |
| C | -3.675268000 | 0.687390000 | -1.399091000 |
| C | -4.994473000 | 1.110699000 | -1.223496000 |
| C | -5.751976000 | 0.621452000 | -0.158117000 |
| C | -5.189864000 | -0.297929000 | 0.728989000 |
| Te | -1.103457000 | -0.914530000 | -0.818285000 |
| C | -0.412169000 | -0.049869000 | 1.091938000 |
| O | -1.179005000 | 0.290034000 | 1.957681000 |
| C | 1.135954000 | 0.124449000 | 1.216156000 |
| C | 1.904986000 | -0.985753000 | 0.528420000 |
| C | 2.711012000 | -0.728919000 | -0.606344000 |
| N | 2.757941000 | 0.545094000 | -1.183059000 |
| C | 2.277417000 | 1.656537000 | -0.487484000 |
| C | 1.472955000 | 1.497214000 | 0.660997000 |
| C | 3.470197000 | -1.786164000 | -1.149999000 |
| C | 3.403862000 | -3.065246000 | -0.611102000 |
| C | 2.588947000 | -3.328418000 | 0.490269000 |
| C | 1.855987000 | -2.288357000 | 1.050340000 |
| C | 2.587603000 | 2.966076000 | -0.910671000 |
| C | 2.084188000 | 4.072745000 | -0.237272000 |
| C | 1.265197000 | 3.913342000 | 0.882152000 |
| C | 0.974567000 | 2.627968000 | 1.322425000 |
| O | 1.468211000 | 0.260069000 | 2.619940000 |
| O | 0.862909000 | -0.796728000 | 3.415414000 |
| C | 3.327506000 | 0.717903000 | -2.513066000 |
| H | 3.249166000 | 3.123442000 | -1.753043000 |
| H | 2.344563000 | 5.067156000 | -0.588622000 |
| H | 0.867710000 | 4.775433000 | 1.407494000 |
| H | 0.350921000 | 2.477030000 | 2.195708000 |
| H | 1.241333000 | -2.465678000 | 1.923589000 |
| H | 2.531230000 | -4.326159000 | 0.912818000 |
| H | 4.002568000 | -3.856546000 | -1.053284000 |
| H | 4.140776000 | -1.603518000 | -1.979988000 |
| H | 2.824243000 | 1.542867000 | -3.018846000 |
| H | 4.406767000 | 0.921443000 | -2.494683000 |
| H | 3.150327000 | -0.181360000 | -3.103927000 |
| H | -3.450007000 | -1.458783000 | 1.236903000 |
| H | -5.774891000 | -0.681461000 | 1.560305000 |
| H | -6.776308000 | 0.955540000 | -0.019608000 |
| H | -5.424927000 | 1.830065000 | -1.914687000 |
| H | -3.086280000 | 1.086424000 | -2.219254000 |
| H | -0.025538000 | -0.413998000 | 3.540663000 |

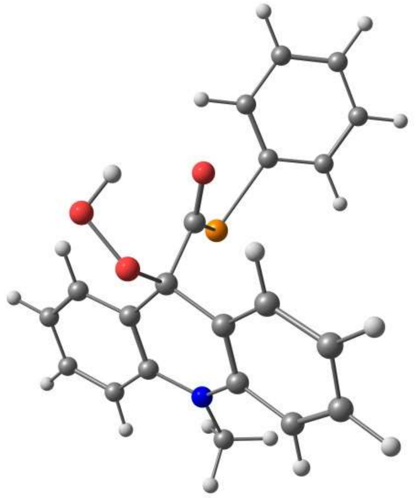

| R = Te | | | |
|---|---|---|---|
| **Structure no. 4**<br>*E* = –239.746242242 Ha | | | |
| Atom | X | Y | Z |
| C | 1.199037000 | -1.287224000 | 0.000000000 |
| C | 0.000000000 | -0.540690000 | 0.000000000 |
| C | -1.199037000 | -1.287224000 | 0.000000000 |
| C | -1.201319000 | -2.682991000 | 0.000000000 |
| C | 0.000000000 | -3.399270000 | 0.000000000 |
| C | 1.201319000 | -2.682991000 | 0.000000000 |
| Te | 0.000000000 | 1.609792000 | 0.000000000 |
| H | -2.145083000 | -0.752178000 | 0.000000000 |
| H | 2.145083000 | -0.752178000 | 0.000000000 |
| H | -2.151326000 | -3.217499000 | 0.000000000 |
| H | 0.000000000 | -4.487469000 | 0.000000000 |
| H | 2.151326000 | -3.217498000 | 0.000000000 |

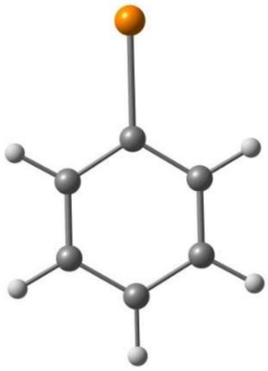

**R = Te**

**Structure no. 6**

*E* = –1023.71267584 Ha

| Atom | X | Y | Z |
|---|---|---|---|
| C | 3.734260000 | 0.780390000 | -1.135010000 |
| C | 3.090615000 | -0.212319000 | -0.386001000 |
| C | 3.794953000 | -0.895515000 | 0.612722000 |
| C | 5.132087000 | -0.580615000 | 0.862362000 |
| C | 5.775545000 | 0.402730000 | 0.109074000 |
| C | 5.076353000 | 1.080209000 | -0.890969000 |
| Te | 1.049899000 | -0.699944000 | -0.801707000 |
| C | 0.360494000 | -0.029172000 | 1.168927000 |
| O | 1.134585000 | 0.215114000 | 2.062978000 |
| C | -1.190297000 | 0.124116000 | 1.416403000 |
| C | -1.624061000 | 1.450986000 | 0.801574000 |
| C | -2.247030000 | 1.494687000 | -0.463451000 |
| N | -2.503722000 | 0.304777000 | -1.160179000 |
| C | -2.576138000 | -0.906673000 | -0.456280000 |
| C | -1.964793000 | -1.029399000 | 0.811279000 |
| C | -2.616218000 | 2.746168000 | -0.992367000 |
| C | -2.351454000 | 3.919259000 | -0.291337000 |
| C | -1.733487000 | 3.875684000 | 0.958931000 |
| C | -1.382987000 | 2.639492000 | 1.494440000 |
| C | -3.268210000 | -2.016312000 | -0.980472000 |
| C | -3.333145000 | -3.212557000 | -0.272394000 |
| C | -2.732330000 | -3.331266000 | 0.981844000 |
| C | -2.061785000 | -2.234110000 | 1.513577000 |
| O | -1.353127000 | 0.133908000 | 2.823359000 |
| C | -2.905139000 | 0.358297000 | -2.556777000 |
| H | -3.786075000 | -1.938523000 | -1.928481000 |
| H | -3.875210000 | -4.050699000 | -0.701170000 |
| H | -2.790778000 | -4.261266000 | 1.538151000 |
| H | -1.606397000 | -2.285145000 | 2.496374000 |
| H | -0.920504000 | 2.579247000 | 2.473591000 |
| H | -1.532611000 | 4.788718000 | 1.510148000 |
| H | -2.647736000 | 4.871075000 | -0.723057000 |
| H | -3.138202000 | 2.806919000 | -1.939492000 |
| H | -2.624818000 | -0.573352000 | -3.050803000 |
| H | -3.984952000 | 0.515745000 | -2.692184000 |
| H | -2.368950000 | 1.168000000 | -3.054599000 |
| H | 3.191504000 | 1.323882000 | -1.902180000 |
| H | 5.570380000 | 1.849934000 | -1.477375000 |
| H | 6.817955000 | 0.640508000 | 0.301083000 |
| H | 5.671403000 | -1.109920000 | 1.642942000 |
| H | 3.304434000 | -1.667074000 | 1.196178000 |
| H | -0.463190000 | 0.268930000 | 3.193744000 |

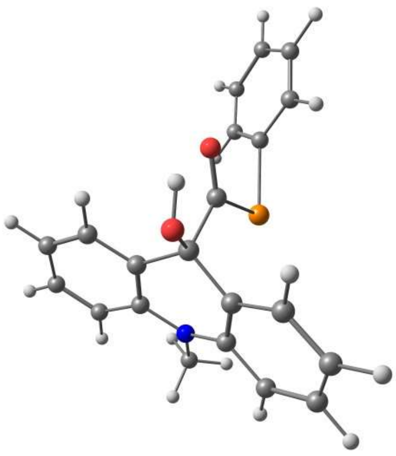

| R = NH | | | |
| --- | --- | --- | --- |
| **Structure no. 1**<br>*E* = –995.042190249 Ha | | | |
| Atom | X | Y | Z |
| C | 3.983850000 | -0.383659000 | -0.841398000 |
| C | 3.251501000 | 0.012528000 | 0.285383000 |
| C | 3.909955000 | 0.351376000 | 1.475278000 |
| C | 5.298594000 | 0.294023000 | 1.542142000 |
| C | 6.038038000 | -0.101793000 | 0.425929000 |
| C | 5.375407000 | -0.437120000 | -0.754691000 |
| N | 1.829882000 | 0.100708000 | 0.293932000 |
| C | 0.949836000 | -0.261921000 | -0.676232000 |
| O | 1.217949000 | -0.708892000 | -1.783532000 |
| C | -0.516275000 | -0.089663000 | -0.304170000 |
| C | -1.094867000 | 1.193144000 | -0.253189000 |
| C | -2.490151000 | 1.322634000 | 0.070234000 |
| N | -3.224646000 | 0.193429000 | 0.364323000 |
| C | -2.707729000 | -1.075443000 | 0.192656000 |
| C | -1.319428000 | -1.235564000 | -0.143613000 |
| C | -0.340062000 | 2.373876000 | -0.533918000 |
| C | -0.929021000 | 3.611012000 | -0.510808000 |
| C | -2.307182000 | 3.722006000 | -0.211765000 |
| C | -3.074739000 | 2.612841000 | 0.072648000 |
| C | -3.514829000 | -2.229793000 | 0.338132000 |
| C | -2.962890000 | -3.483462000 | 0.182274000 |
| C | -1.592125000 | -3.655052000 | -0.121893000 |
| C | -0.791374000 | -2.555245000 | -0.285558000 |
| C | -4.600119000 | 0.343213000 | 0.884085000 |
| H | -4.131699000 | 2.745515000 | 0.254542000 |
| H | -2.779528000 | 4.699015000 | -0.220306000 |
| H | -0.350592000 | 4.499297000 | -0.738470000 |
| H | 0.707358000 | 2.273125000 | -0.792351000 |
| H | 0.248191000 | -2.669093000 | -0.566045000 |
| H | -1.185013000 | -4.652628000 | -0.242596000 |
| H | -3.602039000 | -4.354697000 | 0.283354000 |
| H | -4.574414000 | -2.148835000 | 0.534121000 |
| H | -4.672581000 | 1.268079000 | 1.449436000 |
| H | -5.329363000 | 0.346352000 | 0.069567000 |
| H | -4.814909000 | -0.472256000 | 1.569622000 |
| H | 3.336806000 | 0.657124000 | 2.347928000 |
| H | 5.800607000 | 0.558099000 | 2.467309000 |
| H | 7.120844000 | -0.147022000 | 0.477069000 |
| H | 5.943453000 | -0.743985000 | -1.627215000 |
| H | 3.474007000 | -0.642440000 | -1.758300000 |
| H | 1.430297000 | 0.428906000 | 1.163651000 |

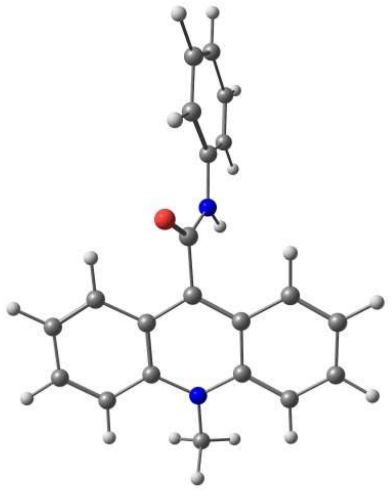

**R = NH**

**Structure no. 2**

*E* = –1146.19582424 Ha

| Atom | X | Y | Z |
|---|---|---|---|
| C | 0.427008000 | -2.323719000 | 1.107160000 |
| C | 1.018929000 | -1.198068000 | 0.507799000 |
| C | 1.995006000 | -1.396079000 | -0.499763000 |
| C | 2.406786000 | -2.715336000 | -0.790318000 |
| C | 1.824833000 | -3.806328000 | -0.157111000 |
| C | 0.812265000 | -3.620905000 | 0.783887000 |
| C | 0.651624000 | 0.191284000 | 0.996718000 |
| C | 1.368847000 | 1.289496000 | 0.229259000 |
| C | 2.299359000 | 1.002319000 | -0.790972000 |
| N | 2.526473000 | -0.316604000 | -1.208413000 |
| C | 3.001108000 | 2.073911000 | -1.380530000 |
| C | 2.760506000 | 3.385982000 | -0.989868000 |
| C | 1.825766000 | 3.670548000 | 0.005446000 |
| C | 1.149172000 | 2.619504000 | 0.612955000 |
| C | -0.888107000 | 0.491490000 | 0.994458000 |
| O | -1.379907000 | 1.281707000 | 1.782604000 |
| O | 1.156575000 | 0.424200000 | 2.338566000 |
| O | 0.470163000 | -0.427343000 | 3.297450000 |
| C | 3.271000000 | -0.560587000 | -2.438168000 |
| N | -1.573292000 | -0.138185000 | -0.013289000 |
| C | -2.929764000 | -0.008807000 | -0.379137000 |
| C | -3.371363000 | -0.765464000 | -1.476517000 |
| C | -4.694746000 | -0.688544000 | -1.899595000 |
| C | -5.598303000 | 0.143046000 | -1.235746000 |
| C | -5.157144000 | 0.892574000 | -0.144973000 |
| C | -3.834502000 | 0.827159000 | 0.293242000 |
| H | 3.762247000 | 1.884825000 | -2.126344000 |
| H | 3.320453000 | 4.187548000 | -1.462938000 |
| H | 1.638098000 | 4.693279000 | 0.315114000 |
| H | 0.432866000 | 2.806571000 | 1.404294000 |
| H | -0.351956000 | -2.161936000 | 1.842539000 |
| H | 0.335809000 | -4.469162000 | 1.264173000 |
| H | 2.167300000 | -4.806398000 | -0.406590000 |
| H | 3.205300000 | -2.892243000 | -1.499080000 |
| H | 3.103043000 | 0.261137000 | -3.134703000 |
| H | 4.351079000 | -0.664837000 | -2.271033000 |
| H | 2.901481000 | -1.468350000 | -2.915727000 |
| H | -3.494001000 | 1.404887000 | 1.140315000 |
| H | -5.850587000 | 1.542277000 | 0.381369000 |
| H | -6.631173000 | 0.204326000 | -1.564204000 |
| H | -5.017968000 | -1.280823000 | -2.750726000 |
| H | -2.671705000 | -1.414441000 | -1.998616000 |
| H | 1.161179000 | -1.092883000 | 3.454638000 |
| H | -1.038056000 | -0.799626000 | -0.558892000 |

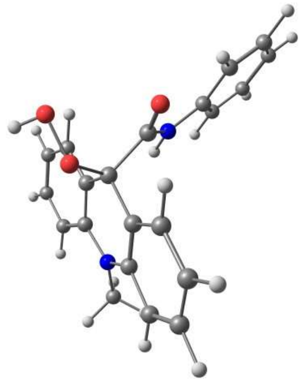

**R = NH**

**Structure no. 4**

*E* = –475.635065838 Ha

| Atom | X | Y | Z |
|---|---|---|---|
| C | -1.205093000 | 0.259174000 | 0.000000000 |
| C | 0.000000000 | 1.067518000 | 0.000000000 |
| C | 1.217134000 | 0.278160000 | 0.000000000 |
| C | 1.215187000 | -1.105164000 | 0.000000000 |
| C | 0.020466000 | -1.852412000 | 0.000000000 |
| C | -1.182463000 | -1.128312000 | 0.000000000 |
| N | 0.064416000 | 2.395719000 | 0.000000000 |
| H | 2.156402000 | 0.829887000 | 0.000000000 |
| H | -2.165453000 | 0.780130000 | 0.000000000 |
| H | 2.172952000 | -1.631243000 | 0.000000000 |
| H | 0.029689000 | -2.940028000 | 0.000000000 |
| H | -2.132514000 | -1.668681000 | 0.000000000 |
| H | -0.903369000 | 2.746120000 | 0.000000000 |

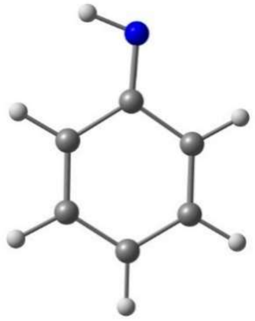

| R = NH | | | | |
|---|---|---|---|---|
| **Structure no. 6**<br>*E* = –1071.08223898 Ha | | | | |
| Atom | X | Y | Z | 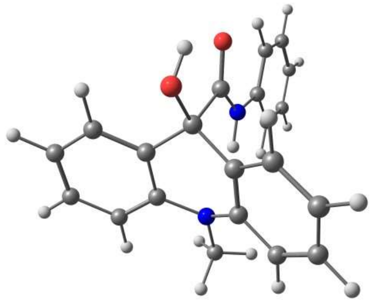 |
| C | 1.706569000 | 2.418117000 | -1.297183000 | |
| C | 1.437738000 | 1.244530000 | -0.595370000 | |
| C | 1.612636000 | 1.210492000 | 0.802018000 | |
| C | 2.045982000 | 2.367449000 | 1.466387000 | |
| C | 2.288376000 | 3.539362000 | 0.749905000 | |
| C | 2.123730000 | 3.571111000 | -0.633445000 | |
| C | 0.938389000 | -0.000139000 | -1.326449000 | |
| C | 1.438131000 | -1.244361000 | -0.595128000 | |
| C | 1.613126000 | -1.209983000 | 0.802246000 | |
| N | 1.331895000 | 0.000263000 | 1.492743000 | |
| C | 2.047033000 | -2.366627000 | 1.466790000 | |
| C | 2.289719000 | -3.538629000 | 0.750548000 | |
| C | 2.124849000 | -3.570748000 | -0.632766000 | |
| C | 1.707229000 | -2.418034000 | -1.296694000 | |
| C | -0.636648000 | -0.000358000 | -1.384714000 | |
| O | -1.176750000 | -0.000385000 | -2.490087000 | |
| O | 1.362220000 | -0.000223000 | -2.667482000 | |
| C | 1.378376000 | 0.000469000 | 2.947929000 | |
| N | -1.286279000 | -0.000587000 | -0.193345000 | |
| C | -2.676837000 | -0.000429000 | 0.059540000 | |
| C | -3.648309000 | -0.000596000 | -0.952397000 | |
| C | -4.998427000 | -0.000462000 | -0.601654000 | |
| C | -5.399916000 | -0.000169000 | 0.734208000 | |
| C | -4.428895000 | -0.000013000 | 1.736985000 | |
| C | -3.077677000 | -0.000149000 | 1.404631000 | |
| H | 2.221831000 | -2.356655000 | 2.535716000 | |
| H | 2.625647000 | -4.423582000 | 1.283048000 | |
| H | 2.322338000 | -4.480634000 | -1.190481000 | |
| H | 1.590932000 | -2.404007000 | -2.374388000 | |
| H | 1.590392000 | 2.403813000 | -2.374883000 | |
| H | 2.321048000 | 4.480939000 | -1.191318000 | |
| H | 2.623881000 | 4.424571000 | 1.282250000 | |
| H | 2.220543000 | 2.357815000 | 2.535354000 | |
| H | 0.857729000 | -0.882118000 | 3.323403000 | |
| H | 2.401915000 | 0.000837000 | 3.350376000 | |
| H | 0.857206000 | 0.882863000 | 3.323163000 | |
| H | -2.325129000 | -0.000022000 | 2.189527000 | |
| H | -4.721190000 | 0.000215000 | 2.782942000 | |
| H | -6.454440000 | -0.000084000 | 0.991650000 | |
| H | -5.744070000 | -0.000594000 | -1.391443000 | |
| H | -3.340731000 | -0.000799000 | -1.988135000 | |
| H | 0.529379000 | -0.000092000 | -3.184231000 | |
| H | -0.691400000 | -0.000335000 | 0.629902000 | |

| R = PH | | | |
|---|---|---|---|
| **Structure no. 1**<br>*E* = −1281.60578101 Ha | | | |
| Atom | X | Y | Z |
| C | 4.127088000 | 1.001038000 | -0.611699000 |
| C | 3.299225000 | 0.682260000 | 0.477088000 |
| C | 3.751661000 | -0.221049000 | 1.452428000 |
| C | 5.015421000 | -0.797885000 | 1.335933000 |
| C | 5.829196000 | -0.489625000 | 0.243665000 |
| C | 5.382250000 | 0.405494000 | -0.729700000 |
| P | 1.679913000 | 1.520125000 | 0.625132000 |
| C | 0.718028000 | 0.675839000 | -0.761818000 |
| O | 1.182791000 | 0.547574000 | -1.876249000 |
| C | -0.690223000 | 0.208588000 | -0.451901000 |
| C | -1.725807000 | 1.136345000 | -0.230076000 |
| C | -3.021675000 | 0.658331000 | 0.169742000 |
| N | -3.215110000 | -0.693823000 | 0.363842000 |
| C | -2.253570000 | -1.622916000 | 0.018933000 |
| C | -0.949014000 | -1.175886000 | -0.385033000 |
| C | -1.536478000 | 2.540157000 | -0.418002000 |
| C | -2.566361000 | 3.424232000 | -0.224264000 |
| C | -3.839016000 | 2.938910000 | 0.154040000 |
| C | -4.070042000 | 1.592884000 | 0.346962000 |
| C | -2.522182000 | -3.012522000 | 0.047991000 |
| C | -1.533534000 | -3.915547000 | -0.281828000 |
| C | -0.236658000 | -3.489205000 | -0.649805000 |
| C | 0.046708000 | -2.149033000 | -0.704051000 |
| C | -4.484148000 | -1.162630000 | 0.958174000 |
| H | -5.070382000 | 1.267652000 | 0.594825000 |
| H | -4.661240000 | 3.635759000 | 0.280963000 |
| H | -2.414128000 | 4.486545000 | -0.377843000 |
| H | -0.566370000 | 2.899948000 | -0.739379000 |
| H | 1.026621000 | -1.810054000 | -1.015818000 |
| H | 0.522510000 | -4.220533000 | -0.903316000 |
| H | -1.764890000 | -4.975757000 | -0.267260000 |
| H | -3.507463000 | -3.384746000 | 0.290079000 |
| H | -4.867177000 | -0.402901000 | 1.633863000 |
| H | -5.224626000 | -1.379407000 | 0.183518000 |
| H | -4.294744000 | -2.055084000 | 1.548849000 |
| H | 3.124169000 | -0.470921000 | 2.303205000 |
| H | 5.362361000 | -1.490468000 | 2.096515000 |
| H | 6.812050000 | -0.941713000 | 0.155110000 |
| H | 6.015034000 | 0.650445000 | -1.576827000 |
| H | 3.793212000 | 1.705943000 | -1.366027000 |
| H | 1.114586000 | 0.792905000 | 1.704994000 |

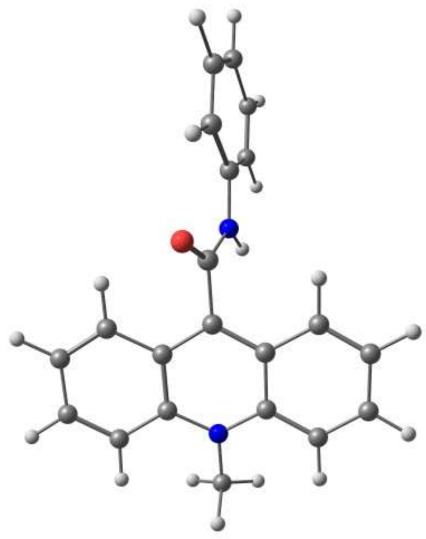

| R = PH | | | |
|---|---|---|---|
| **Structure no. 2**<br>$E$ = –1281.60578101 Ha | | | |
| Atom | X | Y | Z |
| C | -3.756188000 | -1.510813000 | -0.168238000 |
| C | -2.815159000 | -0.741113000 | -0.869776000 |
| C | -3.244520000 | 0.416295000 | -1.536550000 |
| C | -4.586368000 | 0.795544000 | -1.501950000 |
| C | -5.513441000 | 0.031452000 | -0.791510000 |
| C | -5.093762000 | -1.118682000 | -0.121566000 |
| P | -1.072843000 | -1.323975000 | -0.967708000 |
| C | -0.510968000 | -1.020398000 | 0.809739000 |
| O | -1.203338000 | -1.394561000 | 1.739729000 |
| C | 0.879069000 | -0.360488000 | 1.045012000 |
| C | 1.933862000 | -0.880111000 | 0.083812000 |
| C | 2.555443000 | -0.028012000 | -0.858069000 |
| N | 2.138826000 | 1.302254000 | -1.011738000 |
| C | 1.337000000 | 1.913874000 | -0.044394000 |
| C | 0.684746000 | 1.143593000 | 0.941629000 |
| C | 3.598231000 | -0.543851000 | -1.653214000 |
| C | 3.978392000 | -1.877608000 | -1.557274000 |
| C | 3.337154000 | -2.731765000 | -0.662149000 |
| C | 2.328742000 | -2.224041000 | 0.150350000 |
| C | 1.154518000 | 3.312352000 | -0.027919000 |
| C | 0.320557000 | 3.913237000 | 0.908230000 |
| C | -0.349153000 | 3.146642000 | 1.862330000 |
| C | -0.152415000 | 1.770856000 | 1.873145000 |
| O | 1.259335000 | -0.519483000 | 2.435940000 |
| O | 1.188260000 | -1.909721000 | 2.858068000 |
| C | 2.579716000 | 2.067042000 | -2.170309000 |
| H | 1.688521000 | 3.940385000 | -0.729372000 |
| H | 0.204469000 | 4.993101000 | 0.893310000 |
| H | -1.003091000 | 3.612931000 | 2.591478000 |
| H | -0.643078000 | 1.153252000 | 2.616604000 |
| H | 1.847113000 | -2.863383000 | 0.878843000 |
| H | 3.623446000 | -3.775596000 | -0.587755000 |
| H | 4.785850000 | -2.243547000 | -2.184784000 |
| H | 4.136234000 | 0.103786000 | -2.333561000 |
| H | 1.817388000 | 2.801396000 | -2.432667000 |
| H | 3.530021000 | 2.591803000 | -2.003041000 |
| H | 2.694927000 | 1.398832000 | -3.024305000 |
| H | -3.441068000 | -2.410017000 | 0.350981000 |
| H | -5.809885000 | -1.717781000 | 0.433333000 |
| H | -6.557154000 | 0.330118000 | -0.761569000 |
| H | -4.904749000 | 1.693881000 | -2.023087000 |
| H | -2.529459000 | 1.028318000 | -2.079047000 |
| H | 0.225408000 | -1.973689000 | 3.010822000 |
| H | -0.490810000 | -0.173836000 | -1.556258000 |

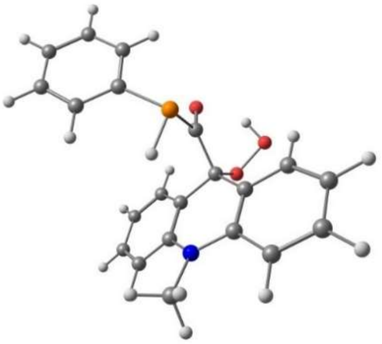

| R = PH | | | |
|---|---|---|---|
| **Structure no. 4**<br>$E$ = –573.614541130 Ha | | | |
| Atom | X | Y | Z |
| C | -1.201510000 | -0.185798000 | 0.000000000 |
| C | 0.000000000 | 0.582890000 | 0.000000000 |
| C | 1.199484000 | -0.191065000 | 0.000000000 |
| C | 1.197356000 | -1.579832000 | 0.000000000 |
| C | -0.003832000 | -2.305165000 | 0.000000000 |
| C | -1.200914000 | -1.576831000 | 0.000000000 |
| P | 0.093148000 | 2.390918000 | 0.000000000 |
| H | 2.153317000 | 0.335049000 | 0.000000000 |
| H | -2.153794000 | 0.340997000 | 0.000000000 |
| H | 2.149223000 | -2.112640000 | 0.000000000 |
| H | -0.005571000 | -3.392764000 | 0.000000000 |
| H | -2.154572000 | -2.106835000 | 0.000000000 |
| H | -1.329325000 | 2.607235000 | 0.000000000 |

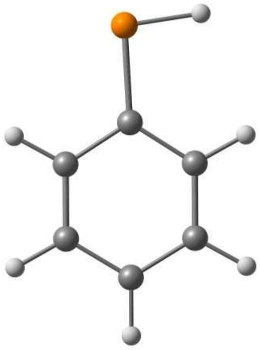

| R = PH | | | |
|---|---|---|---|
| **Structure no. 6** *E* = –1357.64293927 Ha | | | |
| Atom | X | Y | Z |
| C | 0.155033000 | 2.045870000 | -1.888151000 |
| C | 0.794914000 | 1.214380000 | -0.967185000 |
| C | 1.293277000 | 1.758537000 | 0.235276000 |
| C | 1.137870000 | 3.138020000 | 0.468336000 |
| C | 0.480441000 | 3.944876000 | -0.456672000 |
| C | -0.017062000 | 3.405386000 | -1.642338000 |
| C | 0.932670000 | -0.273408000 | -1.277344000 |
| C | 2.050131000 | -0.894022000 | -0.452478000 |
| C | 2.503713000 | -0.284280000 | 0.736550000 |
| N | 1.936416000 | 0.927173000 | 1.167816000 |
| C | 3.537137000 | -0.906598000 | 1.462062000 |
| C | 4.084186000 | -2.111090000 | 1.028586000 |
| C | 3.634750000 | -2.713456000 | -0.146145000 |
| C | 2.628234000 | -2.091855000 | -0.879337000 |
| C | -0.434590000 | -0.983683000 | -0.959600000 |
| O | -1.081334000 | -1.427010000 | -1.893561000 |
| O | 1.166885000 | -0.475115000 | -2.656564000 |
| C | 2.241933000 | 1.432705000 | 2.496271000 |
| P | -1.003333000 | -1.301414000 | 0.801115000 |
| C | -2.775538000 | -0.822388000 | 0.674925000 |
| C | -3.667755000 | -1.675188000 | 0.006787000 |
| C | -5.024925000 | -1.361821000 | -0.061264000 |
| C | -5.511857000 | -0.207856000 | 0.554653000 |
| C | -4.633060000 | 0.639309000 | 1.231261000 |
| C | -3.272001000 | 0.338580000 | 1.286306000 |
| H | 3.939530000 | -0.437902000 | 2.351462000 |
| H | 4.880356000 | -2.568261000 | 1.608946000 |
| H | 4.066320000 | -3.647922000 | -0.489715000 |
| H | 2.279272000 | -2.519280000 | -1.812954000 |
| H | -0.196080000 | 1.605350000 | -2.814812000 |
| H | -0.523836000 | 4.033685000 | -2.367534000 |
| H | 0.375309000 | 5.006215000 | -0.250926000 |
| H | 1.556316000 | 3.593102000 | 1.357528000 |
| H | 2.301325000 | 0.597154000 | 3.195599000 |
| H | 3.185586000 | 1.995371000 | 2.541639000 |
| H | 1.433031000 | 2.084837000 | 2.829540000 |
| H | -2.594417000 | 1.015088000 | 1.799333000 |
| H | -5.004027000 | 1.541490000 | 1.709118000 |
| H | -6.570565000 | 0.029716000 | 0.508402000 |
| H | -5.703486000 | -2.024908000 | -0.589927000 |
| H | -3.299546000 | -2.578439000 | -0.468962000 |
| H | 0.359096000 | -0.911597000 | -2.990473000 |
| H | -0.498530000 | -0.117809000 | 1.390318000 |

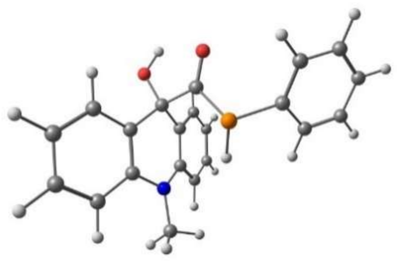

| R = AsH | | | |
|---|---|---|---|
| **Structure no. 1**<br>*E* = –3174.02151596 Ha | | | |
| Atom | X | Y | Z |
| C | 3.473450000 | 0.680713000 | -1.212221000 |
| C | 3.190453000 | -0.434580000 | -0.412517000 |
| C | 4.116812000 | -0.843946000 | 0.555945000 |
| C | 5.308377000 | -0.140149000 | 0.728295000 |
| C | 5.587800000 | 0.966717000 | -0.074388000 |
| C | 4.671591000 | 1.374428000 | -1.046521000 |
| As | 1.563073000 | -1.464537000 | -0.587153000 |
| C | 0.568711000 | -0.479291000 | 0.866390000 |
| O | 1.079418000 | -0.239365000 | 1.935722000 |
| C | -0.847856000 | -0.067205000 | 0.540265000 |
| C | -1.154616000 | 1.302470000 | 0.410719000 |
| C | -2.469886000 | 1.683597000 | -0.025266000 |
| N | -3.392975000 | 0.706221000 | -0.340895000 |
| C | -3.149723000 | -0.630302000 | -0.097430000 |
| C | -1.842774000 | -1.042951000 | 0.333776000 |
| C | -2.789081000 | 3.060058000 | -0.111703000 |
| C | -1.836570000 | 4.011319000 | 0.187861000 |
| C | -0.527243000 | 3.648542000 | 0.579023000 |
| C | -0.195470000 | 2.323105000 | 0.691187000 |
| C | -4.158981000 | -1.611048000 | -0.252664000 |
| C | -3.877160000 | -2.939735000 | -0.013231000 |
| C | -2.590049000 | -3.361339000 | 0.391354000 |
| C | -1.598538000 | -2.431051000 | 0.568525000 |
| C | -4.674120000 | 1.103472000 | -0.960611000 |
| H | -5.168681000 | -1.334778000 | -0.521345000 |
| H | -4.669438000 | -3.672781000 | -0.125710000 |
| H | -2.397584000 | -4.411475000 | 0.579730000 |
| H | -0.617845000 | -2.742364000 | 0.908232000 |
| H | 0.796429000 | 2.032433000 | 1.012899000 |
| H | 0.203810000 | 4.417304000 | 0.802687000 |
| H | -2.106246000 | 5.060848000 | 0.127940000 |
| H | -3.784662000 | 3.386068000 | -0.377239000 |
| H | -5.018733000 | 0.307759000 | -1.615231000 |
| H | -5.430604000 | 1.313750000 | -0.199752000 |
| H | -4.515796000 | 1.983686000 | -1.577967000 |
| H | 2.768336000 | 1.004646000 | -1.972096000 |
| H | 4.889273000 | 2.231994000 | -1.675792000 |
| H | 6.520164000 | 1.507822000 | 0.052941000 |
| H | 6.019718000 | -0.462070000 | 1.482179000 |
| H | 3.910244000 | -1.710730000 | 1.176920000 |
| H | 0.904941000 | -0.647963000 | -1.682569000 |

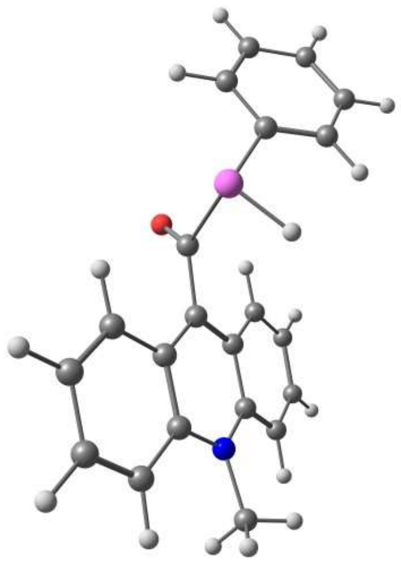

| R = AsH | | | | |
|---|---|---|---|---|
| **Structure no. 2**<br>*E* = −3325.18494918 Ha | | | | |
| Atom | X | Y | Z | |
| C | 1.552862000 | 2.429297000 | 1.502510000 | 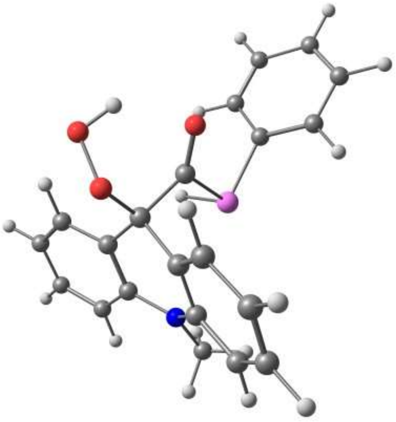 |
| C | 1.609710000 | 1.271973000 | 0.718769000 | |
| C | 2.115524000 | 1.340329000 | -0.596989000 | |
| C | 2.568414000 | 2.578767000 | -1.085354000 | |
| C | 2.508170000 | 3.718020000 | -0.288530000 | |
| C | 2.002334000 | 3.651316000 | 1.010214000 | |
| C | 1.041861000 | -0.044292000 | 1.241155000 | |
| C | 1.685322000 | -1.232701000 | 0.537911000 | |
| C | 2.199609000 | -1.071736000 | -0.766785000 | |
| N | 2.152531000 | 0.187645000 | -1.387509000 | |
| C | 2.761321000 | -2.178237000 | -1.426437000 | |
| C | 2.807420000 | -3.422384000 | -0.804497000 | |
| C | 2.311838000 | -3.581863000 | 0.488685000 | |
| C | 1.757849000 | -2.487631000 | 1.150441000 | |
| C | -0.491086000 | 0.063671000 | 0.962835000 | |
| O | -1.276441000 | 0.361970000 | 1.835091000 | |
| O | 1.292914000 | -0.015277000 | 2.653447000 | |
| O | 0.456811000 | -0.975041000 | 3.357348000 | |
| C | 2.287231000 | 0.293333000 | -2.831030000 | |
| As | -1.062033000 | -0.108443000 | -0.974176000 | |
| C | -2.969375000 | -0.090217000 | -0.603794000 | |
| C | -3.638413000 | 1.139268000 | -0.654568000 | |
| C | -5.005240000 | 1.221991000 | -0.388275000 | |
| C | -5.724799000 | 0.068988000 | -0.073506000 | |
| C | -5.070378000 | -1.162711000 | -0.020702000 | |
| C | -3.701450000 | -1.238190000 | -0.277028000 | |
| H | 2.993446000 | 2.646801000 | -2.079556000 | |
| H | 2.869266000 | 4.661683000 | -0.687043000 | |
| H | 1.953822000 | 4.539782000 | 1.631102000 | |
| H | 1.149073000 | 2.354195000 | 2.504756000 | |
| H | 1.362083000 | -2.597753000 | 2.151814000 | |
| H | 2.352610000 | -4.548563000 | 0.980204000 | |
| H | 3.248276000 | -4.263421000 | -1.331744000 | |
| H | 3.191943000 | -2.060698000 | -2.413695000 | |
| H | 1.761510000 | 1.186102000 | -3.174118000 | |
| H | 3.331668000 | 0.345731000 | -3.169174000 | |
| H | 1.810644000 | -0.571503000 | -3.296296000 | |
| H | -3.089856000 | 2.043600000 | -0.906368000 | |
| H | -5.506395000 | 2.184708000 | -0.429716000 | |
| H | -6.790170000 | 0.129570000 | 0.128974000 | |
| H | -5.623570000 | -2.064345000 | 0.226401000 | |
| H | -3.205034000 | -2.202877000 | -0.224939000 | |
| H | -0.387882000 | -0.482347000 | 3.354454000 | |
| H | -0.880673000 | -1.610701000 | -0.941945000 | |

| R = AsH | | | | |
|---|---|---|---|---|
| **Structure no. 4**<br>*E* = −2466.02342961 Ha | | | | |
| Atom | X | Y | Z | |
| C | 0.161862000 | -2.508448000 | 0.000000000 | 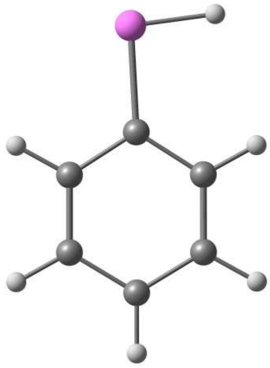 |
| C | -0.591265000 | -1.338574000 | 0.000000000 | |
| C | 0.000000000 | -0.045559000 | 0.000000000 | |
| C | 1.422826000 | -0.043876000 | 0.000000000 | |
| C | 2.178375000 | -1.210035000 | 0.000000000 | |
| C | 1.562446000 | -2.470064000 | 0.000000000 | |
| As | -0.951503000 | 1.612964000 | 0.000000000 | |
| H | 1.938837000 | 0.915763000 | 0.000000000 | |
| H | -1.676677000 | -1.412612000 | 0.000000000 | |
| H | 3.266731000 | -1.139833000 | 0.000000000 | |
| H | 2.151144000 | -3.384640000 | 0.000000000 | |
| H | -0.351769000 | -3.470758000 | 0.000000000 | |
| H | -2.334120000 | 0.963613000 | 0.000000000 | |

| R = AsH | | | |
| --- | --- | --- | --- |
| **Structure no. 6** $E$ = −3250.06294234 Ha | | | |
| Atom | X | Y | Z |
| C | 0.702670000 | 0.821614000 | -0.128643000 |
| C | 0.337936000 | -0.076496000 | 0.887204000 |
| C | 1.355638000 | -0.663752000 | 1.654154000 |
| C | 2.692946000 | -0.341960000 | 1.418354000 |
| C | 3.041195000 | 0.554688000 | 0.411477000 |
| C | 2.037446000 | 1.134166000 | -0.369149000 |
| As | -1.549133000 | -0.582512000 | 1.057270000 |
| C | -1.746572000 | -0.412126000 | 3.023991000 |
| O | -0.851095000 | -0.730986000 | 3.783195000 |
| C | -3.139971000 | -0.052434000 | 3.673339000 |
| C | -3.761045000 | 1.190285000 | 3.060052000 |
| C | -4.532437000 | 1.075782000 | 1.884138000 |
| N | -4.742586000 | -0.203419000 | 1.324030000 |
| C | -4.758402000 | -1.324802000 | 2.179528000 |
| C | -4.016520000 | -1.275481000 | 3.376868000 |
| C | -5.059254000 | 2.229875000 | 1.295525000 |
| C | -4.818076000 | 3.482823000 | 1.866295000 |
| C | -4.067620000 | 3.597284000 | 3.032378000 |
| C | -3.549610000 | 2.443398000 | 3.627258000 |
| C | -5.494697000 | -2.474782000 | 1.874373000 |
| C | -5.480916000 | -3.566789000 | 2.744527000 |
| C | -4.755371000 | -3.518828000 | 3.932554000 |
| C | -4.028603000 | -2.367312000 | 4.241345000 |
| O | -2.926800000 | 0.114121000 | 5.051415000 |
| C | -5.199981000 | -0.333591000 | -0.048220000 |
| H | -6.100907000 | -2.518071000 | 0.976788000 |
| H | -6.059521000 | -4.450165000 | 2.490446000 |
| H | -4.751853000 | -4.365224000 | 4.611682000 |
| H | -3.462753000 | -2.294359000 | 5.163207000 |
| H | -2.977534000 | 2.497710000 | 4.546753000 |
| H | -3.885379000 | 4.569670000 | 3.478531000 |
| H | -5.238443000 | 4.366140000 | 1.394222000 |
| H | -5.681758000 | 2.161142000 | 0.410744000 |
| H | -4.870507000 | -1.297840000 | -0.441938000 |
| H | -6.291723000 | -0.263590000 | -0.155768000 |
| H | -4.740956000 | 0.451726000 | -0.652861000 |
| H | -0.069862000 | 1.282662000 | -0.740709000 |
| H | 2.291912000 | 1.834582000 | -1.158910000 |
| H | 4.084465000 | 0.800030000 | 0.236333000 |
| H | 3.466359000 | -0.799964000 | 2.029627000 |
| H | 1.099341000 | -1.355464000 | 2.446150000 |
| H | -1.998917000 | -0.158207000 | 5.209278000 |
| H | -2.059833000 | 0.842622000 | 0.844020000 |

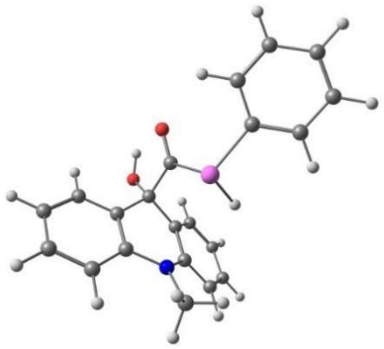

| R = $SiH_2$ | | | |
|---|---|---|---|
| **Structure no. 1** <br> *E* = –1230.35595605 Ha | | | |
| Atom | X | Y | Z |
| C | -3.914392000 | -0.991816000 | -0.743446000 |
| C | -3.305857000 | -0.796389000 | 0.512611000 |
| C | -3.957589000 | 0.015727000 | 1.459986000 |
| C | -5.186829000 | 0.606095000 | 1.165215000 |
| C | -5.777761000 | 0.400994000 | -0.082559000 |
| C | -5.140285000 | -0.395645000 | -1.036802000 |
| Si | -1.678270000 | -1.606506000 | 0.933357000 |
| C | -0.442517000 | -1.262983000 | -0.546332000 |
| O | -0.665983000 | -1.749053000 | -1.643082000 |
| C | 0.804601000 | -0.408165000 | -0.353231000 |
| C | 2.054485000 | -1.027774000 | -0.169166000 |
| C | 3.201997000 | -0.215652000 | 0.131530000 |
| N | 3.053343000 | 1.150734000 | 0.256035000 |
| C | 1.866063000 | 1.783511000 | -0.051403000 |
| C | 0.700412000 | 0.994382000 | -0.340370000 |
| C | 2.216769000 | -2.443328000 | -0.274075000 |
| C | 3.445654000 | -3.026050000 | -0.104342000 |
| C | 4.570777000 | -2.213627000 | 0.168203000 |
| C | 4.461625000 | -0.843836000 | 0.285007000 |
| C | 1.762337000 | 3.194877000 | -0.097087000 |
| C | 0.552799000 | 3.791036000 | -0.386554000 |
| C | -0.608416000 | 3.025703000 | -0.642895000 |
| C | -0.532365000 | 1.657015000 | -0.626408000 |
| C | 4.195550000 | 1.955772000 | 0.736906000 |
| H | 5.357263000 | -0.264248000 | 0.458075000 |
| H | 5.548081000 | -2.672865000 | 0.276709000 |
| H | 3.561840000 | -4.100188000 | -0.194492000 |
| H | 1.354334000 | -3.050276000 | -0.521212000 |
| H | -1.409866000 | 1.061548000 | -0.852618000 |
| H | -1.546093000 | 3.521087000 | -0.867941000 |
| H | 0.499501000 | 4.874142000 | -0.429696000 |
| H | 2.628192000 | 3.823684000 | 0.054046000 |
| H | 4.778224000 | 1.366913000 | 1.440367000 |
| H | 4.828345000 | 2.275445000 | -0.095325000 |
| H | 3.823172000 | 2.823700000 | 1.273777000 |
| H | -3.510064000 | 0.182855000 | 2.436280000 |
| H | -5.682752000 | 1.222671000 | 1.908619000 |
| H | -6.735150000 | 0.859251000 | -0.311258000 |
| H | -5.600833000 | -0.557023000 | -2.006611000 |
| H | -3.429562000 | -1.609060000 | -1.495045000 |
| H | -1.106794000 | -1.040924000 | 2.181886000 |
| H | -1.709925000 | -3.087433000 | 1.001100000 |

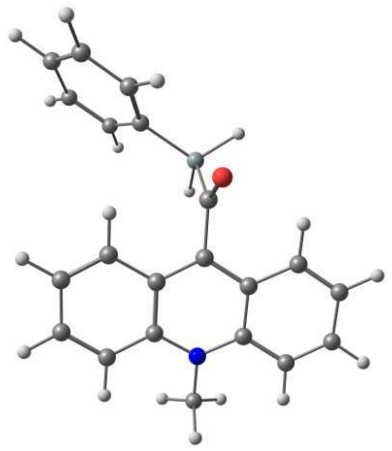

| R = $SiH_2$ | | | |
|---|---|---|---|
| **Structure no. 2** $E$ = –1381.51829108 Ha | | | |
| Atom | X | Y | Z |
| C | 3.552791000 | 0.394276000 | -1.670953000 |
| C | 2.968372000 | -0.666593000 | -0.957021000 |
| C | 3.776633000 | -1.393390000 | -0.062430000 |
| C | 5.123332000 | -1.072487000 | 0.105346000 |
| C | 5.688170000 | -0.019573000 | -0.617596000 |
| C | 4.901173000 | 0.714553000 | -1.505453000 |
| Si | 1.159996000 | -1.101017000 | -1.204884000 |
| C | 0.324835000 | -1.299394000 | 0.542611000 |
| O | 0.794739000 | -2.144421000 | 1.297339000 |
| C | -0.905216000 | -0.453829000 | 0.954827000 |
| C | -0.606984000 | 1.025228000 | 0.813560000 |
| C | -1.426212000 | 1.873722000 | 0.032887000 |
| N | -2.454466000 | 1.352597000 | -0.765725000 |
| C | -2.832724000 | 0.012052000 | -0.666756000 |
| C | -2.076316000 | -0.899423000 | 0.102273000 |
| C | -1.176308000 | 3.260259000 | 0.060689000 |
| C | -0.102813000 | 3.781321000 | 0.775708000 |
| C | 0.748683000 | 2.939631000 | 1.487528000 |
| C | 0.481858000 | 1.573719000 | 1.504905000 |
| C | -3.980987000 | -0.472945000 | -1.328257000 |
| C | -4.330100000 | -1.817255000 | -1.266902000 |
| C | -3.561369000 | -2.723334000 | -0.535805000 |
| C | -2.450284000 | -2.249297000 | 0.151277000 |
| O | -1.351208000 | -0.819182000 | 2.288012000 |
| O | -0.286875000 | -0.651055000 | 3.266325000 |
| C | -3.083296000 | 2.202685000 | -1.769492000 |
| H | -4.626056000 | 0.205117000 | -1.871399000 |
| H | -5.221041000 | -2.152687000 | -1.789865000 |
| H | -3.833455000 | -3.772174000 | -0.485893000 |
| H | -1.858856000 | -2.922514000 | 0.762797000 |
| H | 1.102319000 | 0.911420000 | 2.094892000 |
| H | 1.596082000 | 3.337974000 | 2.035346000 |
| H | 0.061303000 | 4.855002000 | 0.772581000 |
| H | -1.833355000 | 3.944850000 | -0.459828000 |
| H | -3.384357000 | 1.598083000 | -2.625085000 |
| H | -3.963728000 | 2.735119000 | -1.386603000 |
| H | -2.361754000 | 2.934799000 | -2.132779000 |
| H | 2.950232000 | 0.979537000 | -2.360941000 |
| H | 5.335158000 | 1.537162000 | -2.066761000 |
| H | 6.737587000 | 0.229008000 | -0.486905000 |
| H | 5.732266000 | -1.643067000 | 0.800865000 |
| H | 3.344888000 | -2.205787000 | 0.515310000 |
| H | 0.216853000 | -1.467296000 | 3.079875000 |
| H | 0.502142000 | -0.067942000 | -2.042867000 |
| H | 0.992422000 | -2.432838000 | -1.843365000 |

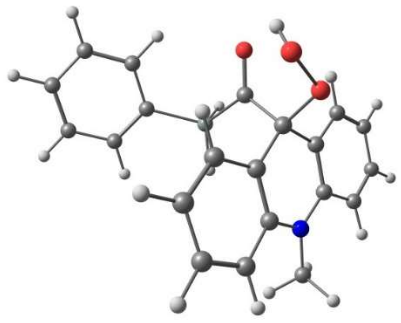

| R = $SiH_2$ | | | |
|---|---|---|---|
| **Structure no. 4** $E$ = –522.347264664 Ha | | | |
| Atom | X | Y | Z |
| C | -1.621157000 | 1.201266000 | -0.000191000 |
| C | -0.225884000 | 1.192996000 | 0.010062000 |
| C | 0.533209000 | -0.000224000 | 0.019042000 |
| C | -0.226195000 | -1.193194000 | 0.010053000 |
| C | -1.621501000 | -1.201074000 | -0.000191000 |
| C | -2.337746000 | 0.000181000 | -0.004083000 |
| Si | 2.475279000 | -0.000031000 | -0.139700000 |
| H | 0.299508000 | -2.147724000 | 0.018427000 |
| H | 0.300134000 | 2.147345000 | 0.018451000 |
| H | -2.157393000 | -2.150629000 | 0.001746000 |
| H | -3.426090000 | 0.000339000 | -0.010215000 |
| H | -2.156817000 | 2.150953000 | 0.001742000 |
| H | 2.740939000 | 1.141328000 | 0.858528000 |
| H | 2.741453000 | -1.140886000 | 0.858965000 |

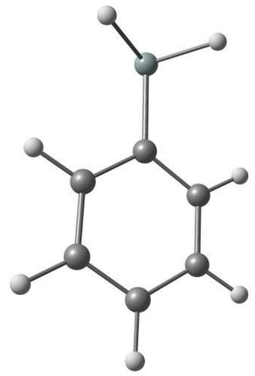

| R = $SiH_2$ | | | | |
|---|---|---|---|---|
| **Structure no. 6**<br>*E* = –1306.39547985 Ha | | | | |
| Atom | X | Y | Z | 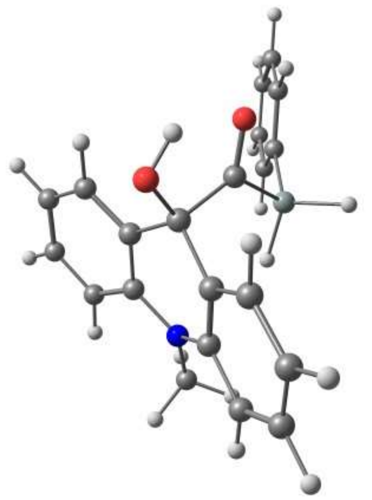 |
| C | 0.031585000 | 1.849429000 | -2.012449000 | |
| C | 0.742633000 | 1.139757000 | -1.042057000 | |
| C | 1.142796000 | 1.789437000 | 0.145528000 | |
| C | 0.820783000 | 3.149945000 | 0.311111000 | |
| C | 0.097338000 | 3.832964000 | -0.662776000 | |
| C | -0.303687000 | 3.188666000 | -1.832674000 | |
| C | 1.058679000 | -0.332220000 | -1.278667000 | |
| C | 2.217027000 | -0.785558000 | -0.399215000 | |
| C | 2.566475000 | -0.074314000 | 0.769337000 | |
| N | 1.864278000 | 1.087841000 | 1.123581000 | |
| C | 3.638915000 | -0.546302000 | 1.549924000 | |
| C | 4.322586000 | -1.705380000 | 1.192937000 | |
| C | 3.973960000 | -2.409938000 | 0.041065000 | |
| C | 2.928999000 | -1.935744000 | -0.747476000 | |
| C | -0.207002000 | -1.175620000 | -0.911184000 | |
| O | -0.722850000 | -1.835974000 | -1.807618000 | |
| O | 1.334091000 | -0.577205000 | -2.641196000 | |
| C | 2.050915000 | 1.671468000 | 2.441362000 | |
| Si | -1.028795000 | -1.300597000 | 0.840478000 | |
| C | -2.852021000 | -0.886219000 | 0.686943000 | |
| C | -3.654323000 | -1.434813000 | -0.331263000 | |
| C | -5.012424000 | -1.128740000 | -0.412313000 | |
| C | -5.595327000 | -0.270093000 | 0.522191000 | |
| C | -4.814834000 | 0.286108000 | 1.535918000 | |
| C | -3.455095000 | -0.018396000 | 1.614757000 | |
| H | 3.964064000 | 0.006119000 | 2.422813000 | |
| H | 5.145109000 | -2.045729000 | 1.815385000 | |
| H | 4.511163000 | -3.309004000 | -0.242879000 | |
| H | 2.654600000 | -2.443287000 | -1.665901000 | |
| H | -0.239273000 | 1.330057000 | -2.925208000 | |
| H | -0.861838000 | 3.721483000 | -2.595595000 | |
| H | -0.137025000 | 4.882129000 | -0.507355000 | |
| H | 1.160876000 | 3.688904000 | 1.186765000 | |
| H | 2.185284000 | 0.874116000 | 3.174146000 | |
| H | 2.914910000 | 2.349232000 | 2.498573000 | |
| H | 1.154245000 | 2.227134000 | 2.720567000 | |
| H | -2.858254000 | 0.428307000 | 2.406105000 | |
| H | -5.262363000 | 0.958624000 | 2.262231000 | |
| H | -6.653595000 | -0.033249000 | 0.457926000 | |
| H | -5.616382000 | -1.559338000 | -1.205972000 | |
| H | -3.209031000 | -2.092668000 | -1.071767000 | |
| H | 0.628828000 | -1.189158000 | -2.932566000 | |
| H | -0.807396000 | -2.716150000 | 1.237018000 | |
| H | -0.398530000 | -0.405235000 | 1.838468000 | |

| R = $GeH_2$ | | | |
|---|---|---|---|
| **Structure no. 1** $E$ = –3015.85885833 Ha | | | |
| Atom | X | Y | Z |
| C | -3.847288000 | -0.587864000 | -1.046257000 |
| C | -3.253335000 | 0.470485000 | -0.342128000 |
| C | -3.888103000 | 0.972882000 | 0.804878000 |
| C | -5.093594000 | 0.423602000 | 1.239024000 |
| C | -5.679015000 | -0.627972000 | 0.529805000 |
| C | -5.055225000 | -1.134506000 | -0.611513000 |
| Ge | -1.609175000 | 1.248350000 | -0.965583000 |
| C | -0.284296000 | 1.127133000 | 0.544690000 |
| O | -0.504047000 | 1.741629000 | 1.571274000 |
| C | 0.988608000 | 0.315874000 | 0.387179000 |
| C | 0.925362000 | -1.088732000 | 0.328618000 |
| C | 2.124481000 | -1.836165000 | 0.071598000 |
| N | 3.305532000 | -1.162235000 | -0.164338000 |
| C | 3.407196000 | 0.205386000 | -0.008528000 |
| C | 2.223733000 | 0.978459000 | 0.256010000 |
| C | 2.060893000 | -3.251073000 | 0.076026000 |
| C | 0.857691000 | -3.889107000 | 0.291700000 |
| C | -0.336218000 | -3.163402000 | 0.510886000 |
| C | -0.300116000 | -1.793289000 | 0.535574000 |
| C | 4.653688000 | 0.871620000 | -0.091528000 |
| C | 4.716593000 | 2.241920000 | 0.049398000 |
| C | 3.555378000 | 3.017336000 | 0.274027000 |
| C | 2.337584000 | 2.397683000 | 0.379372000 |
| C | 4.493318000 | -1.923781000 | -0.604342000 |
| H | 5.572989000 | 0.320841000 | -0.230548000 |
| H | 5.683892000 | 2.730893000 | -0.005835000 |
| H | 3.634232000 | 4.093757000 | 0.376493000 |
| H | 1.445077000 | 2.976400000 | 0.579774000 |
| H | -1.206620000 | -1.233698000 | 0.739057000 |
| H | -1.268825000 | -3.690790000 | 0.676699000 |
| H | 0.833999000 | -4.974028000 | 0.303513000 |
| H | 2.951187000 | -3.850443000 | -0.050765000 |
| H | 5.093778000 | -1.302685000 | -1.263607000 |
| H | 5.092059000 | -2.245910000 | 0.251786000 |
| H | 4.173675000 | -2.788418000 | -1.179116000 |
| H | -3.365970000 | -0.986836000 | -1.935206000 |
| H | -5.512067000 | -1.948560000 | -1.165886000 |
| H | -6.620962000 | -1.050409000 | 0.865932000 |
| H | -5.579066000 | 0.818520000 | 2.125999000 |
| H | -3.437038000 | 1.788560000 | 1.362666000 |
| H | -1.058596000 | 0.489471000 | -2.180848000 |
| H | -1.688498000 | 2.749829000 | -1.238225000 |

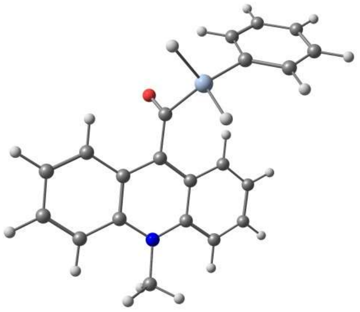

| R = $GeH_2$ | | | |
|---|---|---|---|
| **Structure no. 2**<br>*E* = –3167.02306322 Ha | | | |
| Atom | X | Y | Z |
| C | 3.234267000 | 0.700190000 | -0.958299000 |
| C | 2.840763000 | -0.630274000 | -0.755987000 |
| C | 3.814541000 | -1.571731000 | -0.393361000 |
| C | 5.146474000 | -1.191689000 | -0.228165000 |
| C | 5.525215000 | 0.136046000 | -0.433411000 |
| C | 4.566403000 | 1.082416000 | -0.798678000 |
| Ge | 0.995021000 | -1.159116000 | -0.969150000 |
| C | 0.128941000 | -1.218586000 | 0.830914000 |
| O | 0.547978000 | -2.014179000 | 1.655023000 |
| C | -1.058453000 | -0.275145000 | 1.118093000 |
| C | -2.214381000 | -0.747480000 | 0.260027000 |
| C | -2.872299000 | 0.108862000 | -0.649079000 |
| N | -2.406461000 | 1.405725000 | -0.877982000 |
| C | -1.383428000 | 1.954509000 | -0.092532000 |
| C | -0.661061000 | 1.158474000 | 0.826797000 |
| C | -4.014230000 | -0.384033000 | -1.315198000 |
| C | -4.444822000 | -1.692556000 | -1.127336000 |
| C | -3.766255000 | -2.551561000 | -0.262328000 |
| C | -2.664662000 | -2.063496000 | 0.429997000 |
| C | -1.041467000 | 3.316405000 | -0.214621000 |
| C | 0.032355000 | 3.852040000 | 0.488975000 |
| C | 0.792999000 | 3.050767000 | 1.337914000 |
| C | 0.433749000 | 1.716015000 | 1.501365000 |
| O | -1.564737000 | -0.474175000 | 2.461596000 |
| O | -0.513012000 | -0.285944000 | 3.450159000 |
| C | -2.927769000 | 2.165134000 | -2.008491000 |
| H | -4.589835000 | 0.262443000 | -1.964600000 |
| H | -5.327515000 | -2.036289000 | -1.658745000 |
| H | -4.099988000 | -3.573236000 | -0.116199000 |
| H | -2.142048000 | -2.695942000 | 1.139785000 |
| H | 0.983402000 | 1.091052000 | 2.193515000 |
| H | 1.640654000 | 3.458656000 | 1.878226000 |
| H | 0.268300000 | 4.905469000 | 0.368982000 |
| H | -1.626831000 | 3.972813000 | -0.845205000 |
| H | -3.223593000 | 1.480902000 | -2.803692000 |
| H | -3.789884000 | 2.789951000 | -1.741247000 |
| H | -2.142851000 | 2.804858000 | -2.412252000 |
| H | 3.532146000 | -2.608175000 | -0.229674000 |
| H | 5.889714000 | -1.931313000 | 0.055500000 |
| H | 6.563068000 | 0.431835000 | -0.309486000 |
| H | 4.855880000 | 2.116925000 | -0.960024000 |
| H | 2.492725000 | 1.444789000 | -1.234802000 |
| H | -0.058233000 | -1.145726000 | 3.354316000 |
| H | 0.280164000 | -0.211480000 | -1.939752000 |
| H | 0.936146000 | -2.597329000 | -1.489781000 |

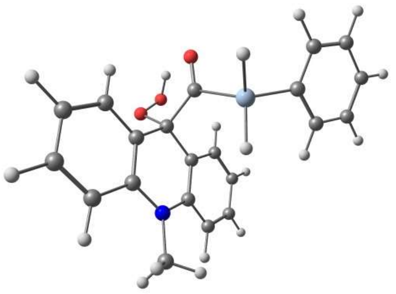

| R = $GeH_2$ | | | |
|---|---|---|---|
| **Structure no. 4**<br>*E* = –2307.81227692 Ha | | | |
| Atom | X | Y | Z |
| C | -1.199266000 | 2.198439000 | 0.000000000 |
| C | -1.202870000 | 0.811348000 | 0.000000000 |
| C | 0.000000000 | 0.044739000 | 0.000000000 |
| C | 1.203486000 | 0.810261000 | 0.000000000 |
| C | 1.201066000 | 2.197392000 | 0.000000000 |
| C | 0.001250000 | 2.927799000 | 0.000000000 |
| Ge | -0.000765000 | -1.836885000 | 0.000000000 |
| H | 2.157388000 | 0.285396000 | 0.000000000 |
| H | -2.157214000 | 0.287284000 | 0.000000000 |
| H | 2.154308000 | 2.727648000 | 0.000000000 |
| H | 0.001747000 | 4.014926000 | 0.000000000 |
| H | -2.152034000 | 2.729534000 | 0.000000000 |
| H | 1.314956000 | -2.602332000 | 0.000000000 |
| H | -1.316670000 | -2.601999000 | 0.000000000 |

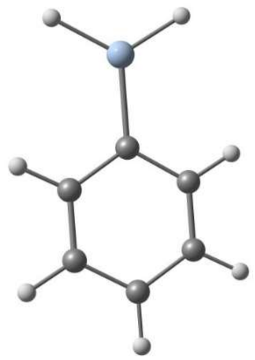

| R = $GeH_2$ | | | | |
|---|---|---|---|---|
| **Structure no. 6** $E$ = –3091.89958062 Ha | | | | |
| Atom | X | Y | Z | |
| C | 3.012001000 | 0.506171000 | -0.620313000 | 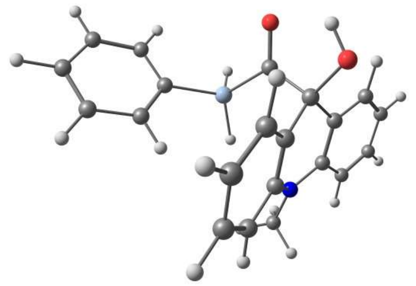 |
| C | 2.649671000 | -0.846486000 | -0.541766000 | |
| C | 3.653988000 | -1.801698000 | -0.333894000 | |
| C | 4.988035000 | -1.415304000 | -0.200642000 | |
| C | 5.335927000 | -0.066346000 | -0.280555000 | |
| C | 4.345121000 | 0.894769000 | -0.490463000 | |
| Ge | 0.805763000 | -1.393181000 | -0.720555000 | |
| C | -0.079161000 | -1.181419000 | 1.053458000 | |
| O | 0.308982000 | -1.904258000 | 1.956210000 | |
| C | -1.188870000 | -0.130726000 | 1.370939000 | |
| C | -0.645939000 | 1.247071000 | 1.019012000 | |
| C | -0.934831000 | 1.844584000 | -0.227467000 | |
| N | -1.773817000 | 1.195089000 | -1.143164000 | |
| C | -2.650857000 | 0.193494000 | -0.698857000 | |
| C | -2.411482000 | -0.465092000 | 0.525871000 | |
| C | -0.376774000 | 3.105402000 | -0.516051000 | |
| C | 0.460886000 | 3.738346000 | 0.399417000 | |
| C | 0.747888000 | 3.145033000 | 1.628799000 | |
| C | 0.184924000 | 1.906803000 | 1.926758000 | |
| C | -3.788935000 | -0.163006000 | -1.446097000 | |
| C | -4.644408000 | -1.167538000 | -1.001498000 | |
| C | -4.402281000 | -1.824430000 | 0.204731000 | |
| C | -3.290569000 | -1.459605000 | 0.959437000 | |
| O | -1.476604000 | -0.232024000 | 2.748352000 | |
| C | -1.831645000 | 1.651272000 | -2.522045000 | |
| H | -4.026218000 | 0.362434000 | -2.363075000 | |
| H | -5.514604000 | -1.422845000 | -1.599245000 | |
| H | -5.071701000 | -2.603058000 | 0.555610000 | |
| H | -3.092439000 | -1.931566000 | 1.915625000 | |
| H | 0.369283000 | 1.431177000 | 2.883851000 | |
| H | 1.394912000 | 3.640125000 | 2.345453000 | |
| H | 0.874686000 | 4.711386000 | 0.150656000 | |
| H | -0.622057000 | 3.611315000 | -1.441828000 | |
| H | -2.103389000 | 0.814444000 | -3.167394000 | |
| H | -2.552612000 | 2.465681000 | -2.681237000 | |
| H | -0.842816000 | 1.996183000 | -2.829907000 | |
| H | 2.248014000 | 1.262886000 | -0.778349000 | |
| H | 4.610173000 | 1.946366000 | -0.552413000 | |
| H | 6.374599000 | 0.235337000 | -0.179610000 | |
| H | 5.756313000 | -2.165873000 | -0.038305000 | |
| H | 3.395854000 | -2.855156000 | -0.269188000 | |
| H | -0.902643000 | -0.953931000 | 3.072699000 | |
| H | 0.116485000 | -0.614496000 | -1.842092000 | |
| H | 0.765319000 | -2.891757000 | -1.010498000 | |

| Structure no. 3<br>*E* = –858.562621852 Ha | | | |
|---|---|---|---|
| Atom | X | Y | Z |
| C | 0.000001668 | 0.000001693 | -0.000001706 |
| C | -0.000001089 | -0.000001851 | 0.000001165 |
| C | -0.000000367 | -0.000001169 | -0.000000734 |
| C | -0.000002814 | 0.000000924 | 0.000001399 |
| C | -0.000000362 | 0.000001022 | -0.000004683 |
| C | 0.000002700 | 0.000001164 | 0.000003988 |
| H | -0.000002124 | -0.000001075 | -0.000000977 |
| H | -0.000000648 | -0.000000525 | 0.000000174 |
| H | -0.000000321 | -0.000000268 | -0.000000218 |
| H | -0.000000691 | 0.000002504 | 0.000001310 |
| C | -0.000017086 | 0.000054670 | 0.000000418 |
| C | 0.000010024 | -0.000005601 | 0.000004297 |
| C | 0.000000008 | 0.000002454 | 0.000003356 |
| N | 0.000000508 | -0.000004494 | -0.000002943 |
| C | 0.000010417 | -0.000023592 | -0.000005269 |
| C | 0.000002427 | 0.000000477 | 0.000001251 |
| C | -0.000002474 | 0.000002539 | -0.000002411 |
| C | 0.000001761 | 0.000000169 | -0.000000460 |
| C | -0.000000665 | -0.000000821 | 0.000001710 |
| C | -0.000000299 | -0.000001818 | -0.000000104 |
| H | -0.000000966 | 0.000001462 | -0.000000044 |
| H | -0.000000352 | 0.000000638 | 0.000000331 |
| H | -0.000000532 | -0.000000897 | 0.000000546 |
| H | -0.000000829 | -0.000002301 | 0.000003179 |
| H | 0.000001748 | -0.000002958 | 0.000000892 |
| H | -0.000001643 | -0.000002553 | -0.000000051 |
| H | -0.000000226 | -0.000002195 | -0.000003133 |
| O | -0.000004137 | 0.000004980 | 0.000005157 |
| O | -0.000005940 | 0.000022784 | 0.000000099 |
| O | 0.000012303 | -0.000045363 | -0.000006540 |

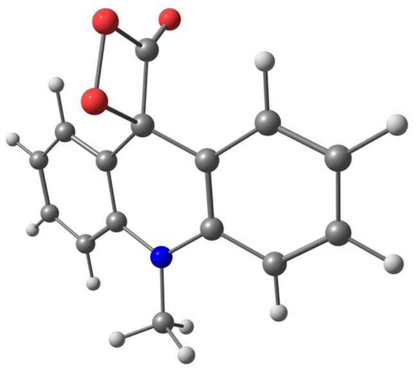

| Structure no. 5<br>*E* = –670.130875334 Ha | | | |
|---|---|---|---|
| Atom | X | Y | Z |
| C | -0.000001670 | -0.000000453 | -0.000001193 |
| C | 0.000008374 | -0.000005822 | -0.000001257 |
| C | 0.000002869 | 0.000003676 | -0.000003162 |
| C | -0.000004216 | 0.000000572 | 0.000006893 |
| C | -0.000005048 | 0.000000891 | -0.000003333 |
| C | 0.000004889 | -0.000001754 | -0.000002000 |
| N | -0.000012637 | 0.000017666 | -0.000007600 |
| C | 0.000006051 | -0.000000082 | 0.000013394 |
| C | -0.000000121 | 0.000000061 | -0.000000647 |
| C | 0.000007382 | -0.000003957 | 0.000003724 |
| C | 0.000002584 | 0.000000596 | -0.000005419 |
| C | -0.000003789 | -0.000000740 | -0.000003533 |
| C | 0.000000399 | 0.000001187 | 0.000002142 |
| C | -0.000007344 | -0.000001750 | 0.000004438 |
| O | -0.000006036 | 0.000000369 | -0.000002764 |
| H | 0.000004494 | -0.000002110 | -0.000007293 |
| H | 0.000001227 | -0.000000156 | 0.000000932 |
| H | 0.000001465 | 0.000000453 | 0.000000462 |
| H | -0.000001085 | 0.000000180 | 0.000002749 |
| H | 0.000001397 | 0.000000188 | -0.000001976 |
| H | 0.000001000 | 0.000000064 | 0.000001035 |
| H | 0.000000113 | 0.000000643 | 0.000000580 |
| H | -0.000002946 | -0.000001652 | 0.000004237 |
| C | 0.000015838 | -0.000020655 | 0.000006897 |
| H | -0.000003439 | 0.000010208 | -0.000003254 |
| H | -0.000003390 | 0.000004209 | -0.000004274 |
| H | -0.000006362 | -0.000001834 | 0.000000222 |

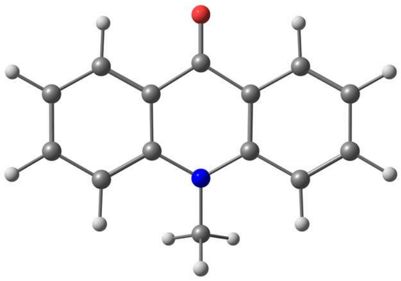

| Structure no. 5* <br> *E* = –669.953518683 Ha | | | |
|---|---|---|---|
| Atom | X | Y | Z |
| C | 0.001468800 | 0.001306003 | -0.014311619 |
| C | 0.004149509 | -0.000735113 | 0.018325511 |
| C | -0.007929979 | 0.000236431 | 0.003322763 |
| C | 0.006366228 | -0.000302096 | -0.001943657 |
| C | 0.002246243 | -0.000461559 | 0.001194686 |
| C | -0.005244037 | -0.001119324 | -0.002114223 |
| C | 0.002234971 | 0.002222086 | 0.001039014 |
| C | -0.001297352 | -0.000930055 | -0.008452168 |
| C | 0.017660061 | -0.003111007 | -0.005658745 |
| N | -0.006147717 | -0.003163889 | -0.003149872 |
| C | 0.001646542 | 0.000527929 | 0.006435157 |
| C | 0.002147453 | -0.000444212 | 0.001369963 |
| C | -0.004625234 | -0.001228109 | -0.003212401 |
| C | -0.011272831 | 0.003546751 | 0.008307965 |
| C | 0.002077794 | 0.004103694 | 0.000763843 |
| O | -0.002882271 | -0.000726284 | -0.001551682 |
| H | 0.000157316 | 0.000901467 | 0.000726183 |
| H | 0.000013596 | -0.000065916 | 0.000470644 |
| H | 0.000088075 | 0.000455664 | 0.000087984 |
| H | -0.000411324 | 0.000239363 | 0.000014109 |
| H | -0.000182535 | 0.000199133 | -0.000392060 |
| H | 0.000158968 | 0.000443207 | -0.000037869 |
| H | 0.000401083 | -0.000134057 | -0.000217245 |
| H | 0.000774354 | 0.000792950 | -0.000369232 |
| H | -0.000473261 | -0.000738933 | 0.000646528 |
| H | 0.000239722 | -0.000864334 | -0.000619243 |
| H | -0.001364177 | -0.000949789 | -0.000674335 |

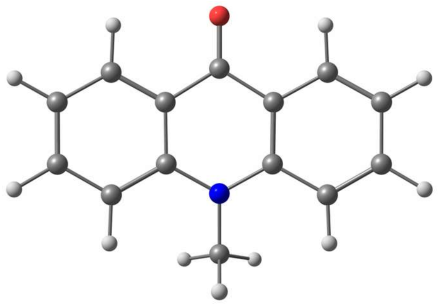

| Structure no. 7 <br> *E* = –783.845241706 Ha | | | |
|---|---|---|---|
| Atom | X | Y | Z |
| C | -0.000003697 | 0.000002446 | -0.000014793 |
| C | 0.000002314 | -0.000000933 | 0.000008333 |
| C | -0.000001217 | -0.000003198 | 0.000002700 |
| C | 0.000004336 | 0.000000328 | 0.000001581 |
| C | 0.000004926 | -0.000001305 | 0.000003524 |
| C | -0.000007755 | -0.000001029 | -0.000007555 |
| N | 0.000007393 | 0.000000704 | 0.000000979 |
| C | -0.000005213 | -0.000002967 | -0.000001330 |
| C | 0.000000330 | 0.000003971 | 0.000003834 |
| C | -0.000011000 | -0.000008618 | -0.000007880 |
| C | -0.000002247 | 0.000003865 | -0.000007202 |
| C | -0.000002250 | 0.000001186 | 0.000006776 |
| C | 0.000003876 | 0.000000304 | 0.000005644 |
| C | 0.000009818 | 0.000004769 | -0.000005549 |
| C | -0.000012508 | 0.000004987 | 0.000019564 |
| O | 0.000003593 | 0.000002240 | 0.000001128 |
| C | -0.000008468 | -0.000005079 | -0.000006976 |
| O | -0.000006529 | -0.000007501 | -0.000010075 |
| H | 0.000001778 | 0.000001712 | 0.000001095 |
| H | -0.000000735 | 0.000001005 | 0.000000100 |
| H | 0.000002823 | 0.000000808 | 0.000000856 |
| H | 0.000009003 | 0.000001827 | -0.000001928 |
| H | 0.000001635 | 0.000000603 | 0.000003297 |
| H | 0.000001306 | -0.000000319 | 0.000001102 |
| H | -0.000001737 | -0.000001334 | -0.000000322 |
| H | -0.000001556 | -0.000001767 | -0.000002097 |
| H | 0.000002183 | 0.000002078 | -0.000006761 |
| H | -0.000000947 | -0.000001576 | 0.000003175 |
| H | 0.000002443 | 0.000002699 | -0.000000949 |
| H | 0.000008102 | 0.000000095 | 0.000009727 |

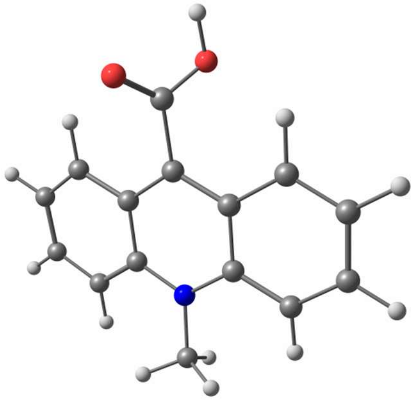

| Structure $CO_2$<br>*E* = –188.580938479 Ha | | | |
|---|---|---|---|
| Atom | X | Y | Z |
| C | -0.000683228 | -0.000000000 | -0.000001192 |
| O | 0.000341891 | 0.000000000 | -0.000158006 |
| O | 0.000341337 | 0.000000000 | 0.000159199 |

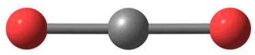

| Structure $H_2O$<br>*E* = –76.4197366208 Ha | | | |
|---|---|---|---|
| Atom | X | Y | Z |
| O | -0.000092754 | 0.000000000 | -0.000065587 |
| H | 0.000031598 | 0.000000000 | 0.000053695 |
| H | 0.000061157 | -0.000000000 | 0.000011892 |

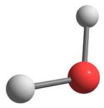

| Structure $OH^-$<br>*E* = –75.7262923857 Ha | | | |
|---|---|---|---|
| Atom | X | Y | Z |
| O | 0.000000000 | 0.000000000 | 0.000008875 |
| H | 0.000000000 | 0.000000000 | -0.000008875 |

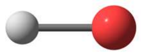

| Structure $OOH^-$<br>*E* = –75.7262923857 Ha | | | |
|---|---|---|---|
| Atom | X | Y | Z |
| O | 0.000158315 | 0.000000000 | -0.000030230 |
| O | -0.000018291 | -0.000000000 | 0.000013673 |
| H | -0.000140024 | -0.000000000 | 0.000016557 |

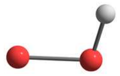

Supplement: Supplementary file 1 [file ijms-24-09468-s001.zip › ijms-2415685-supplementary.pdf]
